# Understanding the value of virtual care technologies: development of a framework in the veterans health administration

**DOI:** 10.3389/fdgth.2026.1677472

**Published:** 2026-05-21

**Authors:** Timothy P. Hogan, Bella Etingen, Nicholas McMahon, Gabriel Escudero, Saige Calkins, Angie Moussa, Cindie Slightam, Donna Zulman, Stephanie L. Shimada, Stephanie A. Robinson, Mark S. Zocchi, Nilesh Shah, Leonie Heyworth, Terry J. Newton

**Affiliations:** 1 eHealth Partnered Evaluation Initiative, Veterans Affairs Bedford Healthcare System, Bedford, MA, United States; 2 Center for Health Optimization and Implementation Research (CHOIR), Veterans Affairs Bedford Healthcare System, Bedford, MA, United States; 3 Department of Health Economics, Systems, and Policy, Peter O’Donnell Jr School of Public Health, University of Texas Southwestern Medical Center, Dallas, TX, United States; 4 Research and Development Service, Dallas Veterans Affairs Medical Center, Dallas, TX, United States; 5 Center of Innovation for Complex Chronic Healthcare (CINCCH), Edward Hines Jr. Veterans Affairs Hospital, Hines, IL, United States; 6 Center for Innovation to Implementation (Ci2i), Veterans Affairs Palo Alto Health Care System, Menlo Park, CA, United States; 7 Division of Primary Care and Population Health, Stanford University School of Medicine, Stanford, CA, United States; 8 Department of Health Law, Policy, and Management, Boston University School of Public Health, Boston, MA, United States; 9 Division of Health Informatics and Implementation Science, Department of Population and Quantitative Health Sciences, University of Massachusetts Medical School, Worcester, MA, United States; 10 The Pulmonary Center, Boston University School of Medicine, Boston, MA, United States; 11 Office of Connected Care, Veterans Health Administration, Washington, DC, United States

**Keywords:** digital health, evaluation, framework, health care and management, health information technologies, value, veterans, virtual care

## Abstract

**Introduction:**

Healthcare systems, including the Veterans Health Administration (VHA), are facing tremendous growth in virtual care technologies that are intended to foster connections between patients, informal caregivers, and healthcare team members. The adoption of some of these virtual care technologies was accelerated by the onset of the COVID-19 pandemic, emphasizing the need to more comprehensively and rigorously assess the impacts of such technologies. An encompassing framework that represents the universe of outcomes relevant to virtual care technologies and can inform related measurement is necessary to understand such impacts in depth. Such a framework can inform how, where, and when these technologies may have the most impact, and by extension, contribute the most value. This article describes the participatory and literature-based development of a Value Framework reflecting the potential value of virtual care technologies for VHA healthcare stakeholder groups and the VHA healthcare system.

**Methods:**

We pursued a combination of participatory co-design approaches involving key stakeholders representing different domains of expertise in VHA and completed a targeted scoping review of 96 prior randomized clinical trials funded by VHA to identify and describe outcomes related to virtual care technologies. Findings from these activities were synthesized and used to inform the Value Framework's organization.

**Results:**

The Framework is comprised of five primary value categories reflective of healthcare's Quintuple Aim: 1. Experiences of Care, 2. Access to and Utilization of Quality Healthcare, 3. Population Health, 4. Costs, and 5. Equity. Each of these primary value categories includes subcategories and sets of distinct outcomes related to the adoption and use of virtual care technologies.

**Discussion:**

Since its development, VHA has adopted this Value Framework to inform efforts in evaluating and communicating the impacts of its portfolio of virtual care technologies. The Value Framework may offer researchers and healthcare organizations a tool that can support the development of a cumulative evidence base regarding the value of virtual care technologies.

## 1 Introduction

Healthcare systems around the globe are increasingly employing diverse technologies to reach and engage the populations they serve. Synchronous video visits bring real-time care into patients’ homes, asynchronous telehealth and remote monitoring platforms offer support for disease management, health promotion and self-management, and web and mobile applications facilitate important transactions such as medication refilling and appointment making. More recent technologies that enhance sharing capabilities, including sensors, wearables, and other devices, offer new data sources that support data-driven treatment decision making and personalized interventions tailored to individual patient needs and situations. Collectively, these capabilities have also changed the day-to-day work of care team members. In many cases, the use of such technologies was accelerated by the COVID-19 pandemic (1–4), during which use of Veterans Health Administration (VHA) video telehealth services saw some 3,000% growth (2, 5) and resulted in significant changes to healthcare delivery and practice. As novel technologies introduce further ways to interact, communicate, and work within the context of healthcare, it is imperative to understand their associated outcomes—or what value they contribute to healthcare consumers, care teams, and the broader systems they are part of. The increased delivery of healthcare services across the care continuum, supported by technology, has the potential to demonstrate profound and lasting value gains. However, understanding these gains requires a fuller and more comprehensive approach than has previously been taken.

The VHA is the largest integrated healthcare system in the United States (US). Like other healthcare systems, VHA views technologies as a contemporary mainstay in the patient care experience and the work of care team members. VHA has increasingly used the term “virtual care” to describe technologies that aim to enhance the accessibility, capacity, quality, and experience of healthcare for patients, their families, and caregivers, wherever they may be located (6). This conceptualization is focused on patient- and healthcare team- facing technologies that have the potential to augment care processes and service delivery, as opposed to back-end systems, electronic health records, and other such technical infrastructure. Common virtual care technologies include, but are not limited to, online patient portals, mobile health applications, synchronous video telehealth, and remote patient monitoring, that have the potential to augment health care processes and service delivery. VHA integrates virtual care into the lives of the patients it serves and the daily work of its staff members, as part of its commitment to providing the right and best care for the US Veteran population. Reflecting the values of a learning healthcare system, VHA recognizes the need for a dedicated effort to assess and understand the value that is accrued (or not accrued) from virtual care technologies. This understanding is essential for achieving continuous quality improvement (7, 8).

An encompassing framework intended to represent the universe of outcomes relevant to virtual care technologies and to inform their measurement can be a critical tool to highlight how, when, and where such technologies may contribute the most value. Prominent healthcare institutions have already posited such frameworks. The National Quality Forum developed a framework to support robust telehealth measurement (9) and conducted literature reviews to identify barriers and facilitators to telehealth use, particularly in rural areas. It also identified corresponding measures to assess telehealth performance (10). Perhaps most closely related to this effort is a foundational report from the American Medical Association that introduces a framework comprised of six overarching virtual care ‘value streams’ and related environmental variables that may influence the realization of value in different contexts (11). Despite the relevance of these works, VHA has unique attributes as a healthcare system, including the vulnerable patient population it serves, its integrated structure, and its use of a capitated system to allocate resources, that has implications for how value can best be conceptualized. These attributes contributed to the need for a dedicated effort to identify and synthesize outcomes that have the potential to relay the value of using virtual care technologies.

This paper proposes a more comprehensive framework for understanding and measuring the value of virtual care technologies for different VHA stakeholder groups and the VHA healthcare system. In the context of this Framework, “value” refers to the benefits and improvements gained from the use of virtual care technologies within a healthcare system. The Value Framework encompasses multiple dimensions, including the quality of care provided, the efficiency and cost-effectiveness of service delivery, the health outcomes achieved, the equity in access and treatment, and the overall experiences of patients, caregivers, and healthcare providers. While there is a large and evolving scientific literature examining outcomes associated with virtual care, studies tend to exhibit tremendous variation in focus and how they conceptualize outcomes of interest. This variation has resulted in an evidence base that, while still valuable, is limited in its capacity to support a cohesive understanding of the value that virtual care technologies may contribute to any given healthcare system, including VHA. The Value Framework we introduce was developed through collaboration between leadership in VHA's Office of Connected Care and members of VHA's Health Systems Research community and includes categories of outcomes and guidance on evaluating such outcomes. The Framework's goal is to support the development of a more cumulative evidence base about the value of virtual care technologies and to facilitate continuous monitoring of VHA's journey towards integrating virtual care into the healthcare services it provides.

## 2 Material and method

As we describe below, our Value Framework development efforts drew upon findings from both participatory co-design activities involving key stakeholders and a targeted scoping review of studies within VHA's Health Systems Research (HSR) portfolio focused on virtual care technologies. This work was reviewed by relevant VHA Institutional Review Boards and designated as program evaluation for quality improvement purposes, exempting it from further oversight (Program Guide 1200.21) (12).

### 2.1 Participatory co-design activities with stakeholders

#### 2.1.1 Stakeholder sample

In late 2021, we launched our participatory co-design activities. We sought to identify stakeholders to participate in a series of elicitation and feedback activities, each described in detail below, to gather perspectives on outcomes related to virtual care technologies of greatest importance to Veterans and the VHA healthcare system. We purposefully selected stakeholders occupying diverse organizational roles, including both clinicians and healthcare leadership, and who had expertise in different domains of virtual care technologies, including: (1) synchronous and asynchronous telehealth platforms, (2) web and mobile platforms, (3) clinical analytics and data management, and (4) implementation and communication strategies to support use of virtual care technologies. Of note, some of these stakeholders were Veterans themselves, enhancing the patient-centeredness of our efforts. We sent invitation emails explaining the goals of the Framework development effort and completed the participatory co-design activities with seven stakeholders (n) over a four-month period.

#### 2.1.2 Activity 1—open-ended outcomes elicitation

Our first participatory activity consisted of a simple email request for open-ended input from our stakeholders. The email included a question intended to generate a preliminary list of outcomes important from their perspectives and associated with use of virtual care technologies. Stakeholders were instructed to open-brainstorm and rapidly list as many ideas as came to mind, without concerns for measurement feasibility, data availability, or other constraints. Two of the authors (TPH, BE) reviewed each stakeholder response and employed inductive thematic analysis to organize responses in broad thematic categories, meeting regularly to establish consensus and discuss any coding disagreements. A copy of the email used for our open-ended outcomes elicitation exercise is included in Supplementary Appendix A.

#### 2.1.3 Activity 2—vignette-driven outcomes elicitation

Following Activity 1, we convened our stakeholder group virtually using MS Teams. In this first group discussion, we presented stakeholders with three outcome vignettes, each addressing a distinct context of patient care. These included: (1) outpatient services, (2) inpatient services, and (3) care delivered in the home/community. We asked our stakeholders to reflect on each vignette and engaged them in a group discussion facilitated by two of the authors (TPH, BE) about the greatest opportunities to improve outcomes in each specific context of care. We recorded the outcomes elicited through these discussions in field notes during the session. Following the session, these authors (TPH, BE) followed the same analytic procedures to incorporate the outcomes into the broad thematic categories generated from Activity 1. Copies of the vignettes used for Activity 2 are included in Supplementary Appendix B.

#### 2.1.4 Activity 3—stakeholder review of identified outcomes

In two additional virtual meetings, the same two authors (TPH, BE) engaged stakeholders in a facilitated discussion of the outcomes related to virtual care technology use identified in Activities 1 and 2. The discussion addressed the nature of the outcomes themselves, the broad thematic categories previously generated, ideas for further organization into more specific subcategories, and any additional outcomes that had not yet been identified. The authors again took field notes to capture the session discussion. Afterwards, the same authors (TPH, BE) used the session feedback to enhance the descriptions of the categories into which outcomes were placed and to establish related subcategories. Category descriptions and subcategories were captured in analytic memos and iteratively refined by our larger team.

#### 2.1.5 Activity 4—stakeholder review of outcome groupings and describing outcomes

We shared the revised outcomes, categories, and descriptions from Activity 3 with the stakeholders in a fifth and final meeting to elicit any last feedback and suggested refinements. The same two authors (TPH, BE) again facilitated the discussion and generated field notes to capture key discussion points. With this feedback, following Activity 4, our larger team proceeded to finalize the characterizations and descriptions of each outcome and their respective categorizations. Reviewing the final categories, we reached consensus that, comparable to other published frameworks (11), the components of the Quintuple Aim in Healthcare (13) would provide a robust yet flexible overall organizational structure for the outcomes and categories identified.

### 2.2 Targeted scoping review of clinical trials

#### 2.2.1 Overview

To complement the insights gathered through our participatory co-design activities with stakeholders and to ensure our emerging Value Framework was adequately grounded in existing evidence, we reviewed select studies funded by VHA's Health Systems Research program. Our review aimed to: (1) identify additional outcomes suitable for incorporation into the Framework beyond those that emerged from our participatory co-design activities, and (2) begin identifying measures used in other studies to examine outcomes in the Framework.

#### 2.2.2 Data sources and review procedures

We used a Python-adapted web-scraping program to identify current and previous research included in VHA's comprehensive database of funded peer-reviewed studies that were related to technology implementation, use, and outcomes. This program utilized a list of approximately 200 keywords developed with input from our stakeholders. We included studies dating from 2011 to the time of our review. We manually reviewed initial results (*n* = 2,006) for relevance, yielding a list of 369 relevant studies. In line with our definition of virtual care technologies, we reviewed these 369 studies to identify those focused on Veteran- and VHA healthcare team member-facing technologies. This review yielded 178 studies, of which 96 were randomized clinical trials designed to examine changes in various outcomes. We reviewed the ClinicalTrials.gov database entries for these *n* = 96 trials to extract information about the outcomes they included and how they were measured. A structured template was created to support the extraction of these outcomes and measures. Three authors (NM, SC, AM) populated the structured templates, extracting the needed information from the 96 trials. When data extraction was completed, the two authors who led the participatory co-design activities (TPH, BE) proceeded to compare the contents of the structured templates to the characterizations and descriptions of outcomes identified during the participatory co-design activities. The goal of this comparative exercise was to determine if the outcomes extracted from the trials were represented in or absent from the Value Framework.

## 3 Results

### 3.1 Organizing the outcomes identified

#### 3.1.1 Participatory co-design activities with stakeholders

We organized the outcomes identified through our participatory co-design activities with stakeholders into five primary value categories reflective of the dimensions of the Triple, Quadruple, and most recently Quintuple Aims for tracking the performance of healthcare systems (13–15). The primary value categories of the Framework are: (1) Experiences of Care, (2) Access to and Utilization of Quality Healthcare, (3) Population Health, (4) Costs, and (5) Equity. Equity is a cross-cutting value category that represents the pursuit of desirable outcomes in all the value categories for all patients. Importantly, in most cases, addressing any one of the identified outcomes could potentially affect one or more of the other outcomes, indicating an interrelatedness among them. This point requires consideration when applying the Value Framework. As with the original rendering of the Triple Aim, and the subsequent Quadruple and Quintuple Aims, the goal of a healthcare system should be to realize gains across all the primary value categories included in this Framework through the use of virtual care, as opposed to gains in one or two of the categories at the exclusion of others (13–15).

The outcomes we identified through our participatory co-design activities with stakeholders are further organized in subcategories under each of the primary value categories of the Framework. These value subcategories represent the various dimensions of each primary value category and provide a lattice to help Framework users select outcomes most applicable to their work. The interrelatedness among outcomes underscores the importance of studying multiple outcomes across the Framework's primary value categories to understand (or at least acknowledge) how changes in value in one category might affect others, reflecting a commitment to pursuing continual improvement across all five primary value categories (14).

#### 3.1.2 Targeted scoping review of clinical trials

When we compared the outcomes identified through our targeted scoping review of clinical trials to those identified through our participatory co-design activities, we found that nearly all the outcomes identified through our review of the 96 trials were already represented; however, we identified seven additional outcomes through our clinical trials review, which we then incorporated into appropriate Value Framework categories.

#### 3.1.3 Depicting the value framework

**Figure 1:**
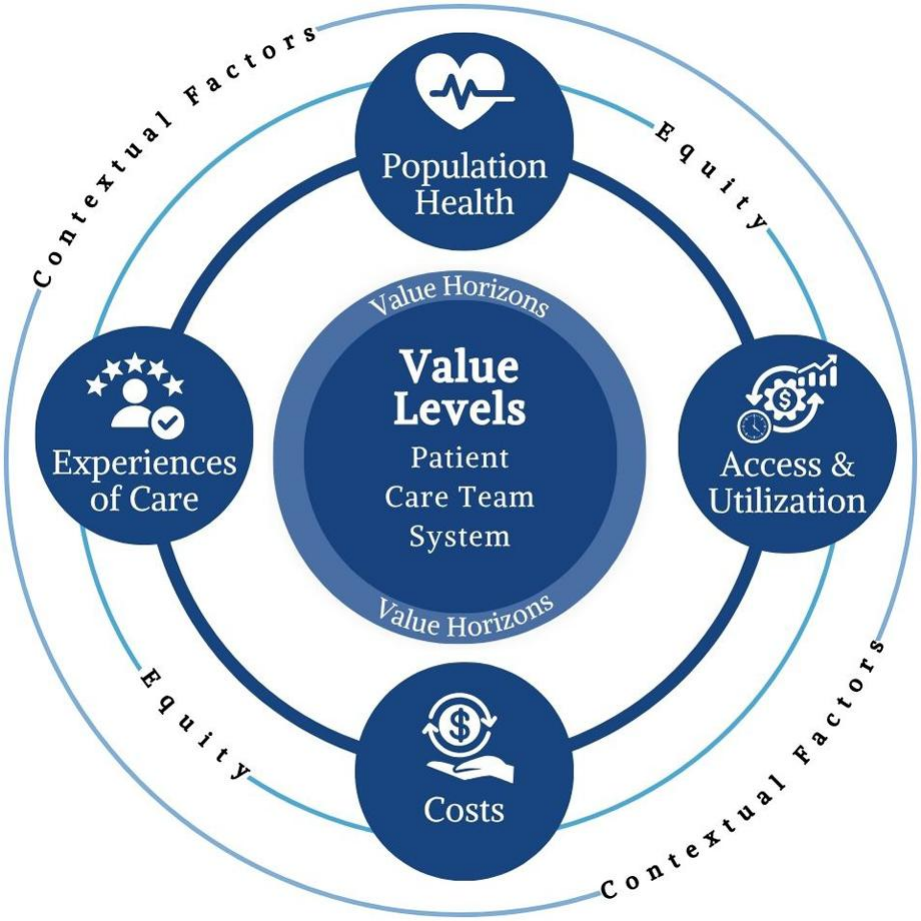
The veterans health administration (VHA) virtual care value framework.

Figure 1 depicts the primary value categories of the Framework and their inter-relatedness. In Table 1, we describe each primary value category of the Framework and its corresponding value subcategories. Beyond the five primary value categories, Figure 1 further highlights additional dimensions that must be considered in relation to each outcome. First, we acknowledge critical determinants that can enhance or hinder the realization of value through virtual care technologies. Around the perimeter of the figure, we note relevant determinants or “contextual factors” that can stem from individuals (e.g., social determinants of health), organizations (e.g., the culture and financial standing of a hospital system), and wider society (e.g., national healthcare policies and laws), and that may ultimately impact outcomes related to the use of virtual care technologies. In the center of the figure, “value levels” refer to the organizational unit in which value may be realized. The levels include the individual patient, care teams, and the overall healthcare system; however, other organizational units may also be appropriate to consider. Finally, around the center of the figure, “value horizons” refer to the timing of when value may be realized, recognizing that virtual care technologies can impact outcomes in both the short term and long term.

**Table 1:** The veterans health administration (VHA) virtual care value framework primary value categories and their subcategories .

Primary value category	Primary value category description	Value subcategories
Experiences of care	Broadly encompasses the perspectives of key stakeholders who interact with or are part of a healthcare system and may use virtual care technologies. It also includes the perspective of the healthcare system itself and the experiences it affords those stakeholders. (16) Additionally, stakeholders’ perspectives about a specific healthcare system and their experiences with virtual care technologies must also be considered. In the original rendering of the Triple Aim, the Experience dimension solely focused on patient experience. (14) In the subsequent Quadruple Aim, the experiences and well-being of healthcare providers were explicitly included. (15) This Value Framework recognizes that different stakeholders may use virtual care technologies, and there is potential for those technologies to impact stakeholder experiences at different organizational levels.	Patient and Informal Caregiver Care Experiences Care Team Member Work Experiences Cross-Cutting Care Experiences Healthcare System Reputation Virtual Care Technology Access and Use
Access to and utilization of quality healthcare	Access to Care refers to the potential value that accompanies the ability of a patient to receive services that meet their needs in a specified period of time, (17) while Utilization of Care refers to patient use of services to promote health and address health problems. (18) Quality in healthcare has been conceptualized as a multidimensional concept, which holds implications for stakeholders at all levels. Of note, access to care has both actual and perceived components. (19) The extent to which patients believe that they can access services when needed is distinct from their actual ability to access those services, with both being important outcomes.	Access to Care Utilization of Care Quality of Care
Population health	The potential value gains afforded by virtual care technologies on clinical outcomes, self-management activities, and health behaviors. In the original rendering of the Triple Aim in Healthcare, this dimension—To Improve the Health of a Population—was considered primary and included a focus on mortality, health, and functional status. (16) This dimension also accounted for disease burden and behavioral and physiological factors, (16) which align naturally with the central focus of many existing virtual care technologies.	Clinical Indices Function Health Promotion and Self-Management
Costs	Encompasses the cost of healthcare services from two perspectives. In the original rendering of the Triple Aim, this dimension was focused on understanding the actual costs of care for an individual, and how to best manage these costs while providing quality care. For this Value Framework, it is important to account for both the perspective of costs to the healthcare system and the costs incurred by the patient and how the use of virtual care technologies could impact those costs.	Costs to the Healthcare System Costs to the Patient
Equity	Equity in healthcare refers to everyone having “a fair and just opportunity to attain their highest level of health.” (20) Equity can only be realized when unfair or addressable differences across groups of people—regardless of race, ethnicity, sexual orientation, demographics or any other characteristics—are eliminated. (21, 22) Comparable to other existing frameworks in the literature, (11) equity is understood to be cross-cutting in this Value Framework. Its focus is on ensuring that outcomes measured in the other primary value categories are equivalent across different patient subgroups. (11)	–

#### 3.1.4 Describing framework value subcategories and associated outcomes

Below, we define the value subcategories that comprise each primary value category of the Framework. A description of each outcome in each value subcategory is included as supplementary material in Supplementary Appendix C.

##### 3.1.4.1 Primary value category: experiences of care

###### 3.1.4.1.1 Patient and informal caregiver care experiences

The value subcategory Patient and Informal Caregiver Care Experiences includes outcomes related to an individual patient's interaction with a healthcare system, as well as the experiences of their informal caregivers. In the case of VHA, patients may receive healthcare services at VHA facilities, community-based facilities, or both. In addition to spanning different healthcare settings, these interactions can also span modalities, ranging from in-person visits to telephone calls and virtual encounters, both synchronous and asynchronous. The outcomes in this value subcategory focus exclusively on the perspectives of patients and their informal caregivers.

###### 3.1.4.1.2 Care team member work experiences

Understanding and improving patient and informal caregiver care experiences is important, but the experiences of care team members must also be considered, especially when evaluating outcomes associated with virtual care technologies. The primary focus here is on outcomes related to how virtual care technologies influence care team members’ work experiences, including how they feel about their work. Professional burnout was a documented concern among providers before the COVID-19 pandemic, which accelerated burnout for many care team members (23). This underscores the importance of creating work environments that promote well-being and retention. Although this value subcategory focuses on care team members, outcomes experienced by the broader healthcare workforce (e.g., administrators, IT) are also included.

###### 3.1.4.1.3 Cross-cutting care experiences

This value subcategory encompasses patients, their informal caregivers, and care team members. Cross-cutting care experiences refer to their interactions, the nature of those interactions, and the results when virtual care technologies are used. At the most fundamental level, this could refer to a single patient and a single care team member interacting, perhaps to share information or decide upon a treatment plan. At broader levels, it could refer to processes of care involving patients interacting with multiple care team members across different settings. Some of these outcomes lend themselves to objective measurement, while others are more subjective but still measurable.

###### 3.1.4.1.5 Healthcare system reputation

Different healthcare systems are known to perform differently based on common measures of care quality. This performance variability is due to a variety of factors, including patient characteristics and routine system performance. It is important for healthcare systems to manage their reputations so that patients see them as a reputable choice for their care needs, care team members see them as a desirable place to work, and the public views them as a valuable community resource.

The value subcategory Healthcare System Reputation includes outcomes related to stakeholder perceptions of a specific healthcare system and of the services that it delivers and how virtual care technologies impact those perceptions. Stakeholders include current recipients of care (e.g., patients, informal caregivers), care providers (e.g., care team members), potential future care recipients and care providers, and broader public opinion.

###### 3.1.4.1.6 Virtual care technology access and use

Although the primary value categories and other value subcategories of this Framework focus on outcomes from use of virtual care technologies, it is also important to consider more proximal outcomes that either precede use of these technologies or are a part of using the technology itself. The Virtual Care Technology Access and Use value subcategory encompasses outcomes that relate to stakeholders’ ability to access and use virtual care technologies as well as their actual use. For the purposes of this value subcategory, “stakeholder” refers to all those who may interact with or use virtual care technologies, including but not limited to, patients, their informal caregivers, care team members, and other staff. “Ability” refers to a continuum of measurable experiences and behaviors that ultimately result in a stakeholder's access to and use of connect care technologies.

##### 3.1.4.2 Primary value category: access to and utilization of quality healthcare

###### 3.1.4.2.1 Access to care

As noted earlier, Access to Care includes both actual and perceived components. The Access to Care value subcategory focuses on actual access to care, measured by more objective data sources rather than stakeholder perceptions. Access to Care refers to the potential value that comes from a patient's ability to receive services that meet their needs in a specified time period (17). The Access to Care value subcategory encompasses outcomes related to the impact of virtual care technologies on the ability of a patient to receive services in a timeframe that aligns with their needs.

###### 3.1.4.2.2 Utilization of care

The value subcategory Utilization of Care encompasses patient use of healthcare services to promote health and address health problems (18). Utilization of care can be influenced by factors at multiple levels, including but not limited to the capacity of a healthcare system and its care team members to provide services, the healthcare needs in a specific community or patient population, and the ability of patients to access services as described in the Access to Care value subcategory.

###### 3.1.4.2.3 Quality of care

Quality in healthcare involves providing effective care and reducing errors in processes and practices (24). The Agency for Healthcare Research and Quality defines quality healthcare as “doing the right thing at the right time in the right way for the right person and having the best results possible,” (25) and acknowledges the six aims articulated by the Institute of Medicine for improving healthcare quality (26). In this way, quality can be conceptualized in relation to healthcare processes and care delivery, as well as healthcare outcomes. Safety is part of the foundation on which quality healthcare is built (26), and quality has implications for individual stakeholders and healthcare systems. Virtual care technologies have the potential to help or hinder the realization of quality in healthcare (27). The Quality of Care value subcategory encompasses outcomes related to the impact of virtual care technologies on the quality of the healthcare services offered by a healthcare system.

##### 3.1.4.3 Primary value category: population health

###### 3.1.4.3.1 Clinical indices

Studies of virtual care technologies have historically been focused on identifying or demonstrating improvements in indicators of clinical status. This focus may have led to missed opportunities to examine other outcomes more closely tied to experiences of care and care delivery processes. Still, demonstrating impacts on clinical indicators is widely recognized as necessary to advance the evidence base on virtual care technologies and to inform how they can best be integrated into clinical care to realize the greatest improvements in patient and population health.

###### 3.1.4.3.2 Function

In addition to clinical indicators, it is important to consider the extent to which an individual can engage in daily activities and life tasks given their health and well-being, and how virtual care technologies may influence such engagement. Limitations to an individual's function may be caused by a variety of factors, including aging, disease, or injury, and can have negative impacts when those factors are not or cannot be appropriately managed (28). Understanding function is relevant at all ages, and is important not only to an individual's daily life, but also to informing how best to deliver care that aligns with what matters most to that individual and the supports they may need to maximize living (29).

###### 3.1.4.3.3 Health promotion and self-management

Virtual care technologies can influence physiological, psychological, and functional outcomes, but they also have the potential to impact individuals’ capacity to improve and manage their own health. Health promotion involves empowering individuals to enhance their overall health (30), while self-management focuses on an individual managing a health condition or disease (31). Both health promotion and self-management are comprised of processes and activities that can be psychological, behavioral, and knowledge-based, and often occur outside of encounters with the healthcare system. Virtual care technologies can potentially contribute to these efforts. Importantly, informal caregivers also play a critical role in supporting patient health promotion and self-management and have their own experiences that must be accounted for. The Health Promotion and Self-Management value subcategory includes outcomes that reflect the processes or activities involved in efforts to improve one's health or manage a disease.

##### 3.1.4.4 Primary value category: costs

###### 3.1.4.4.1 Costs to the healthcare system

Across the US, healthcare costs continue to grow at an unsustainable rate as the resources necessary to provide healthcare services become more expensive and care processes become more complicated. Virtual care technologies have the potential to impact the costs incurred by a healthcare system to deliver its services to patients, and there is optimism that they may enhance cost savings. However, realizing lower costs depends on effective design and implementation. Poorly designed or poorly integrated virtual care technologies can easily exacerbate costs. The Costs to the Healthcare System value subcategory includes outcomes that are likely to be impacted by the increased implementation and use of virtual care technologies.

###### 3.1.4.4.2 Costs to the patient

Similar to the costs incurred by a healthcare system to deliver care, patients incur costs related to receiving care. Some costs are directly tied to care encounters, such as co-pays or transportation and parking for appointments. Other costs may be more indirect, like days missed from work due to health conditions or healthcare appointments. The implementation and use of virtual care technologies have the potential to influence these costs, offering opportunities for savings if the technologies are effectively implemented and meaningfully integrated into healthcare encounters, health promotion, and self-management efforts. The Costs to the Patient value subcategory includes outcomes that reflect both direct, out-of-pocket costs, and potential cost savings for patients.

##### 3.1.4.5 Primary value category: equity

Unlike the other primary value categories in our Value Framework, equity does not include any value subcategories. Instead, equity is conceptualized as the Framework's only cross-cutting primary value category, signifying its central importance to each of the other categories. Similar to other frameworks in the literature, equity in this Value Framework emphasizes the need to monitor outcomes for equivalence across different patient subgroups (11) and is envisioned as a key component of high-reliability healthcare organizations (32). Grounded in the principles of the Triple, Quadruple, and now Quintuple Aims in Healthcare (13–15), which emphasize maximizing all outcomes together, the equity value category emphasizes maximizing the highest level of health for all patients, regardless of who they are.

## 4 Discussion

This paper describes the development of a value framework that delineates outcomes related to the use of virtual care technologies. The Value Framework we developed for the VHA healthcare system is comparable in important ways to other frameworks from leading healthcare institutions (10, 11). Notably, our inductive approach to identifying outcomes through stakeholder-driven participatory approaches, thematic grouping, and a complimentary targeted scoping review of relevant clinical trials resulted in overarching outcome categories best characterized in terms of the Quintuple Aim in healthcare (13–15). These include Population Health, Experiences of Care, Access to and Utilization of Quality Healthcare, Costs, and Equity. Other frameworks by the American Medical Association and National Quality Forum feature similar, yet unique, categorizations of outcomes and value sources (10, 11). This triangulation of outcome categories across frameworks suggests at least some level of saturation in conceptualizing the range of outcomes related to the use of virtual care. Still, many of the outcomes included in this Value Framework are unique, focusing on issues particularly salient to a large integrated healthcare system serving a vulnerable patient population with specific health and social service needs.

The participatory co-design approaches and targeted scoping review of clinical trials that informed the identification of outcomes in this Value Framework also underscored the interdependencies among those outcomes. The implications of those interdependencies for research and evaluation initiatives focused on virtual care technologies are twofold. First, improvements in an outcome in any of the Value Framework's primary value categories may impact outcomes in the other primary categories, creating inherent synergies or tensions. Second, in line with the original conceptualization of the Triple Aim in Healthcare (14), and the subsequent Quadruple and Quintuple Aims (13–15), the goal of any healthcare system should be to realize improvements across all of the Framework's primary value categories through use of virtual care technologies. Realizing gains in only one or a few categories to the exclusion of others reflects a suboptimal pursuit of healthcare system improvements.

### 4.1 Suggestions for value framework use

The most robust research and evaluation initiatives should examine outcomes across the Value Framework's primary value categories. This would likely involve using mixed-methods study designs and gathering data from a variety of primary and secondary sources. Research and evaluation teams must reflect early in their planning and design efforts on the outcomes that they hope to understand or change. The primary value categories and their subcategories provide a lattice to help users select the most applicable outcomes for a particular project. Some general rules of thumb have been noted in other frameworks (33) that can help here, with outcomes selection. These include: (1) the meaningfulness or relevance of the outcome(s) to a particular stakeholder group or care setting; (2) whether the goal is to observe an outcome(s) or intervene to change an outcome(s); (3) the extent to which an outcome(s) could demonstrate variation within the intended targets; (4) whether an additional theory or model is being used to guide the project and what that theory or model suggests as the most meaningful or relevant outcomes; and (5) what sources of data are available for a particular outcome(s).

In addition to selecting outcomes, temporality of when value may be realized (e.g., shorter or longer term) must also be considered. Not all outcomes can be impacted in the short term, and longer time frames may be necessary to evaluate them and, by extension, realize value. Lastly, the organizational unit in which value may be realized must also be considered, recognizing that specific outcomes may be measured at different levels (e.g., the individual patient, the care team, the healthcare system overall) and could be influenced by different contextual factors at each level.

### 4.2 Applications of the value framework

To date, the Value Framework has been disseminated through a variety of channels across the VHA enterprise. It has been integrated into specific Requests for Applications (RFAs) for rapid evaluation projects focused on critical VHA virtual care technologies, and investigators who are part of VHA's intramural research and evaluation programs who are responding to these RFAs are expected to describe the Value Framework outcomes that they are examining in their proposed work. The VHA's Telehealth Effectiveness Coordinating Center (TECC), which is responsible for generating and synthesizing evidence regarding the impacts of VHA's telehealth programs for Congressional reporting purposes has also adopted the Value Framework to guide projects, and to support the synthesis of evidence across projects. The Value Framework has also reached VHA's intramural research and evaluation community through established channels, including workshops at VHA conferences and operational office presentations, as well as the broader non-VHA research community through presentations at scientific meetings. Finally, the more recently established VHA Digital Health Office, which aims to set the gold standard for healthcare delivered through digital health solutions and works to provide the infrastructure and support necessary to deliver effective digital health solutions across VHA, intends to adopt the Value Framework for their cross-cutting evaluation needs. Ideally, the Value Framework will continue to evolve through an iterative process, as it is applied in the aforementioned initiatives, and as the VHA healthcare system works to respond to shifting enterprise-wide priorities and meet both 21st century healthcare demands and the dynamic needs of the Veteran population.

### 4.3 Strengths and limitations

The process we undertook to develop this Value Framework has both strengths and limitations. The outcomes represented in the Framework reflect the input of key organizational stakeholders who possess both clinical and technological expertise, as well as the patient perspective. The participatory co-design activities that we pursued with stakeholders to elicit outcomes relevant to virtual care technologies were grounded in common experiences faced by many patients, increasing the patient-centeredness of these activities. We also complemented our stakeholder participatory co-design activities with a targeted scoping review of recent randomized clinical trials to identify additional outcomes. Finally, we include as Supplementary Material to this paper brief conceptual descriptions and suggested data sources for each outcome identified. This detail represents an advance over previous efforts that have named outcomes but provided limited detail about their scope or meaning.

Conversely, our participatory co-design activities with stakeholders involved a discrete group of subject matter experts. Further representation from other clinical or healthcare management perspectives, as well as gathering additional patient input, may have introduced outcomes not identified here. Although we leveraged recent clinical trials to complement our stakeholder participatory co-design activities, our approach was narrow in scope. By limiting our review to clinical trial study designs, we did not account for other kinds of studies that may have explored outcomes related to the use of virtual care technologies. Lastly, while we began identifying measures used in other studies during our targeted scoping review, this paper does not recommend specific measures for the outcomes included in the Value Framework.

Future work to validate the content of this Value Framework could leverage participatory co-design activities with a broader group of stakeholders who represent a more encompassing range of healthcare organizations and care experiences, as well as a more inclusive review of prior published work to identify outcomes. Further, the consistent use of a common set of core measures might facilitate cross-study comparisons of findings and support the application of advanced analytic techniques and meta-analyses related to specific outcomes. Finally, much can be learned from reports describing early applications of the Value Framework as well as efforts to update the Framework in accordance with the rapidly evolving technology and healthcare landscape.

### 4.4 Conclusions

This Value Framework is the result of a dedicated effort to bring together stakeholder perspectives and existing evidence to advance our understanding of the potential value of virtual care technologies. Its scope is purposefully broad, addressing the variety of virtual care technologies already in use as well as new technologies that may be developed and implemented in the future. Ideally, this Value Framework will encourage further discussion and suggestions for its enhancement. We expect that the Value Framework will evolve iteratively as: (1) healthcare systems like VHA work to meet 21st-century healthcare demands and the dynamic needs of their patient populations, (2) virtual care technologies and the data they offer continue to advance, and (3) research and evaluation teams learn from its application.

## Data Availability

The datasets presented in this article are not readily available because the datasets presented in this article are not readily available because the data were generated as part of a non-research quality improvement evaluation conducted within the United States Department of Veterans Affairs. You may contact the corresponding author to discuss potential availability of specific data elements. Availability of data is governed by applicable regulations concerning availability of data from the United States Department of Veterans Affairs. Inquiries should be directed to Timothy P. Hogan; timothy.hogan@va.gov. Requests to access the datasets should be directed to timothy.hogan@va.gov.
