# Supplementary material for: Understanding the value of virtual care technologies: development of a framework in the veterans health administration
Source: Front Digit Health. 2026 May 21;8:1677472. doi: 10.3389/fdgth.2026.1677472 (PMC13234865; doi:10.3389/fdgth.2026.1677472)
Supplement: Supplementary file 1 [file Datasheet1.pdf]

Supplementary Material

1 Appendix A. Open-Ended Outcomes Elicitation Email

Hi Everyone,

We are writing to request your participation in a simple pre-brainstorming activity in advance of our meeting.

We ask that you each respond to the following question. This will give us a chance to synthesize everyone's responses for discussion at the meeting. Please **respond only to me** (as opposed to reply all) when sending your answers – you can just send a list in email.

Thanks so much in advance and we look forward to the discussion this week. My best,

Tim.

<u>QUESTION:</u> If you could wave a magic wand, what would you like to show that OCC technologies can have a positive impact on?

As you formulate your responses:

- * Remember, there are no right or wrong answers – this is brainstorming!
- * List as many responses as you can. Just as there are no right or wrong answers, there is no such thing as too many answers!
- * Don't constrain your thinking by concerns about data availability, current measurement practices, or what may seem too tough to do!
- * Consider setting yourself a timer for 10 minutes and capture as many ideas as you can!

2 Appendix B. Vignettes for Outcomes Elicitation

OUTCOMES VIGNETTES

- What are the greatest opportunities to improve outcomes for Veterans in their home/community?
- *Robert is a 71-year-old male Vietnam Veteran who has chronic obstructive pulmonary disease (COPD) and co-morbid depression. He lives in rural central Pennsylvania with his wife Cheryl; an important source of social support. He regularly visits VA for his primary and mental health care; however, he sees a pulmonologist in the community to manage his breathing. Robert drives to his appointments and Cheryl accompanies him, but she is increasingly worried about how safe it is for him to do so.*

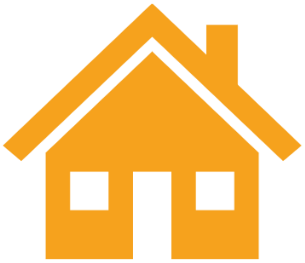

OUTCOMES VIGNETTES

- What are the greatest opportunities to improve outcomes for Veterans receiving outpatient services from VA?
- *Jackie is a 38-year-old female living in greater Cleveland. She served in OEF/OIF/OND and now works as a regional sales consultant. She lives with chronic low back, PTSD, and TBI-related migraines and appreciates that she can receive all her healthcare from VA. She has, however, found it increasingly difficult to attend many of her appointments given her work travel schedule and her responsibilities as a caregiver to her aging parents.*

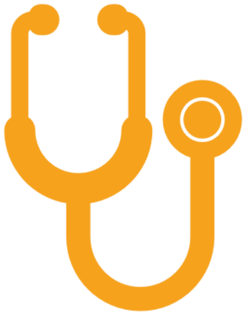

OUTCOMES VIGNETTES

- What are the greatest opportunities to improve outcomes for Veterans receiving inpatient services from VA?
- *Brian is a 48-year-old Veteran of Desert Storm who works as a software developer. He recently sustained a spinal cord injury (T4) while doing roof repairs on his house. He is currently staying at the VA Spinal Cord Injury Center at the St. Louis VAMC for a 3-month period of initial rehabilitation. He hopes to get back to work (and life outside the SCI Center) as soon as possible.*

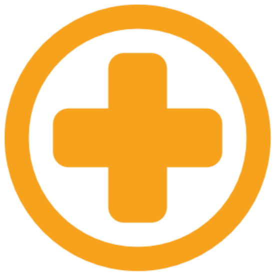

3 Appendix C. Framework Outcomes, Outcome Descriptions, and Possible Data Sources

Patient and Informal Caregiver Care Experiences Outcomes, Descriptions, and Possible Data Sources

Outcomes	Description	Possible Data Source(s)
**Convenience**	Convenience has been defined as "a quality or situation that makes something easy or useful for someone by reducing the amount of work or time required to do something." (1) The integration of virtual care technologies into healthcare services may impact convenience of care for patients, for example, by allowing them to communicate with their care team members and complete transactions with VHA from the comfort of their home or at times that are preferable for them. This outcome represents how and the extent to which use of virtual care technologies impact patient and informal caregiver perceptions of how convenient it is to receive VHA healthcare services.	Self-report
**Engagement**	Patient engagement in care is defined as the extent to which an individual is "actively involved in their health and health care." (2) Engagement is at once both a process and a behavior, and increased engagement has been associated with better health outcomes and lower healthcare costs. (3) This outcome represents how and the extent to which use of virtual care technologies impacts patient and informal caregiver engagement in their health and VHA healthcare services.	Self-report; Administrative data (i.e., service utilization)
**Alignment with patient preferences**	Delivering care in a way that is consistent with patient preferences is foundational to patient-centeredness and VHA's Whole Health model of care. (4) The integration of virtual care technologies into VHA healthcare delivery may impact the ability of VHA to match service delivery with the preferences of patients and their informal caregivers. This outcome represents how and the extent to which use of virtual care technologies impacts the match of VHA healthcare services to patient and informal caregiver preferences for those services.	Self-report
**Ease of transactions**	A healthcare transaction has been defined as information exchange among two parties, and may be facilitated by technology. (5) Some common transactions that patients must complete with the VHA healthcare system include refilling prescription medications and requesting information from one's	Self-report

	medical record. This outcome represents how and the extent to which use of virtual care technologies impacts patient and informal caregiver perceptions of the ease with which they can pursue and complete transactions with VHA, their VHA care team members, or other healthcare systems as needed.	
**Opportunities for patient-to-patient interactions (peer interaction)**	Patients may offer other patients peer support – help founded in shared lived experiences and/or physical or psychological health conditions. (6) Such support can be offered in either a formal way, perhaps through a VHA Peer Support program, or an informal way, perhaps through discussion in a VHA common area. Virtual care technologies may be leveraged to facilitate such patient-to-patient interactions. This outcome represents how and the extent to which use of virtual care technologies facilitates patient engagement with one another in the context of VHA services.	Self-report; Administrative data
**Patient/Caregiver satisfaction with VHA**	In the context of healthcare, satisfaction refers to how happy a patient is with their healthcare services. (7) The integration of virtual care technologies into healthcare service delivery may impact patient satisfaction with care. This outcome represents how and the extent to which use of virtual care technologies impacts patient and informal caregiver perceived happiness with VHA overall.	Self-report; Administrative data (i.e., SHEP survey)
**Inpatient, outpatient, specialty, primary, virtual**	Similar to the assessment of patient and caregiver satisfaction with VHA overall, patients and their informal caregivers may have differing levels of satisfaction with different services provided by VHA and/or different VHA care team members that deliver these services. This outcome represents how and the extent to which use of virtual care technologies impacts patient and informal caregiver perceived happiness with specific VHA services (e.g., inpatient care, outpatient services, specialty care, primary care, virtual care).	

Healthcare Team Member Work Experiences Outcomes, Descriptions, and Possible Data Sources

Outcomes	Description	Possible Data Source(s)
**Workload and workflow**	Healthcare team member workload refers to the amount of work involved in a care team member's job. Measures of workload may encompass such constructs as patient volume, time spent on care delivery, and the	Self-report; Administrative data

	ratio of nursing staff relative to patients. (8) Workflow, on the other hand, refers to “the sequence of physical and mental tasks performed by various people within and between work environments.” (9) The integration of virtual care technologies into service delivery may impact workload (10) as well as clinical workflows, potentially improving or worsening both. This outcome represents how and the extent to which use of virtual care technologies impacts the amount of work that healthcare team members have to do, as well as the way healthcare team members do their work.	
**Burnout**	Work-related burnout is characterized by decreased: emotional resources, perceived success, and feelings of achievement among employees, (11) which can be exacerbated by stress experienced in the workplace. This outcome represents how and the extent to which use of virtual care technologies impacts the experience of burnout among VHA healthcare team members and staff.	Self-report; Administrative data (i.e., AES)
**Job satisfaction**	Employee satisfaction refers to the extent to which an individual likes their job or aspects of that job. (12) Among other factors, the integration of virtual care technologies into the jobs of healthcare team members and staff may impact their job satisfaction. Similarly, the degree to which healthcare team members and staff are satisfied with the virtual care technologies themselves may vary. This outcome represents how and the extent to which use of virtual care technologies impacts VHA healthcare team member and staff satisfaction with their work.	Self-report; Administrative data (i.e., AES)
**Self-efficacy**	Self-efficacy refers to an individual’s perceptions of their ability to perform a task or engage in a behavior. (13) In the context of healthcare, virtual care technologies may impact healthcare team member and staff self-efficacy related to their jobs. This outcome represents how and the extent to which use of virtual care technologies impacts healthcare team member and staff confidence to perform their job duties.	Self-report

**Clinical decision making**	When making a decision about how best to manage a patient, healthcare team members may rely on their experiences, critical thinking skills, available evidence, communication with the patient, and support of colleagues. (14) Clinical decision making may be supported by virtual care technologies. This outcome represents how and the extent to which use of virtual care technologies impacts VHA healthcare team member clinical practice, specifically, their decision-making processes for formulating and/or modifying patient treatment plans.	Self-report

Cross-Cutting Care Experiences Outcomes, Descriptions, and Possible Data Sources

Outcomes	Description	Possible Data Source(s)
**Shared decision-making**	Shared decision-making involves a patient and their healthcare provider making a treatment decision/deciding upon a treatment plan together, based on the best available evidence coupled with patient preferences. (15) Use of virtual care technologies may facilitate shared decision-making. This outcome represents how and to what extent use of virtual care technologies impacts patient and VHA care team member perceptions of the extent to which they engage in shared decision-making together and the quality of the interaction.	Self-report
**Shared goal setting**	Patients and their VHA care team members working together to set health-related goals, and developing treatment plans with those goals in mind, is a core tenet of VHA's Whole Health model. (16) This outcome represents how and to what extent use of virtual care technologies impacts collaborative health-related goal setting among patients and their VHA care team members and the quality of the interaction.	Self-report
**Rapport**	In the context of healthcare, rapport refers to the patient/provider relationship, including how well matched a patient and their provider are and the extent to which they collaborate with one another. (17) This outcome represents how and to what extent use of virtual care technologies impacts perceptions of rapport among patients and VHA care team members.	Self-report
**Communication**	In the context of healthcare, communication refers to strategies used to inform and influence individual and community decisions that affect health. (18) This outcome represents the extent to which use of virtual care technologies impacts the ability of patients and	Self-report

	VHA care team members to communicate with one another, as well as the quality of that communication.	
**Patient-centeredness**	The extent to which health care is patient-centered – that is, centered around the needs and preferences of each individual patient (7) – is a cornerstone of VHA's Whole Health model. (4) This outcome represents how and the extent to which the use of virtual care technologies impacts patient perceptions of the patient-centeredness of their VHA healthcare, as well as VHA care team member ability to deliver patient-centered services.	Self-report; Administrative data (i.e., SHEP)
**Provider-patient interactions**	The way in which care team members interact with patients may impact important aspects of patient care, including their understanding of health conditions, self-management practices, and adherence to treatment plans. (19) Virtual care technologies may be leveraged to facilitate such interactions. This outcome represents how and the extent to which the use of virtual care technologies impacts patient and VHA care team member interactions.	Self-report

VHA Reputation Outcomes, Descriptions, and Possible Data Sources

Outcomes	Description	Possible Data Source(s)
**VHA brand recognition**	Brand recognition refers to an individual's ability to recognize a particular service or organization based on their branding, for example, logo, product packaging, or theme song. (20) VHA has established guidelines regarding its branding, including logos, font types, and colors, used to establish a unified brand across services and products. (21) This outcome represents how and the extent to which use of VHA virtual care technologies impacts VHA brand recognition.	Self-report
**Recognition of VHA's leadership in virtual care**	VHA has been a longstanding, recognized national leader in the development and use of virtual care technologies. This outcome represents the extent to which patients, their informal caregivers, VHA employees, and the public writ large recognize VHA's leadership in this domain.	Self-report
**Trust in the VHA**	Trust is the belief that someone or something is safe, honest, and reliable. (22) VHA assesses trust among patients, their loved ones, and their informal caregivers through their perceptions of three over-arching constructs:	Self-report; Administrative data (e.g., V-Signals)

	effectiveness, ease, and emotion. (23) The integration of virtual care technologies into service delivery may impact individual's trust in VHA. This outcome represents how and the extent to which use of VHA virtual care technologies impacts trust in VHA among patients, their informal caregivers, and the public at large.	
**Choice of VHA as *the* place to receive healthcare services**	Patients have options for where to obtain healthcare services, including from VHA or from community-based providers, through avenues such as the patients Community Care Program (24) or using private insurance. As such, VHA strives to deliver high quality, timely services that patients will choose to utilize. This outcome represents how and the extent to which use of VHA virtual care technologies impacts patient choice to utilize VHA for their care.	Self-report; Administrative data
**Recruitment of care team members**	VHA strives to recruit the best available talent through a range of available programs and initiatives, including internships, leadership opportunities, and hiring incentives. (25) The incorporation of virtual care technologies may influence prospective employee perceptions of the desirability of working for VHA. This outcome represents how and the extent to which use of VHA virtual care technologies impacts VHA's ability to recruit talent.	Self-report
**VHA workforce retention**	Beyond the recruitment of care team members and staff, VHA is focused on retaining the individuals who currently work for the healthcare system, including but not limited to the use of various incentives. (26) The integration of virtual care technologies into care team member and staff workflows may impact employee retention. This outcome represents how and the extent to which use of VHA virtual care technologies impacts retention of care team members and staff in VHA.	Self-report; Administrative data
**Recruiting new patients into care**	Given that patients have options for where to obtain the healthcare services they want/need, VHA strives to provide care in a way that will entice patients who are eligible for VHA services but not yet using them to choose to receive those services from VHA (e.g., as	Self-report; Administrative data

	opposed to community-based care providers). This outcome represents how and the extent to which availability and integration of VHA virtual care technologies into service delivery impacts the ability of VHA to recruit new patients to receive VHA services.	
**Patient retention in VHA care**	In line with patient choice regarding where to initiate receipt of healthcare services, patients who choose to receive services from VHA can, at any point, decide to change where they receive those services to a community-based setting. This outcome represents how and the extent to which use of VHA virtual care technologies impacts retention of patients in VHA care.	Self-report; Administrative data
**Perceived rapport/connectedness with VHA**	Similar to rapport between a patient and their care team members, patients may have a sense of rapport with VHA at large, which may include how well matched a patient feels VHA service delivery is to their needs and preferences. (17) This outcome represents how and the extent to which the integration of VHA virtual care technologies into service delivery impacts patient perceptions of rapport with the VHA healthcare system.	Self-report
**Perceived access to VHA – services and technologies**	Access to care refers to the ability of a patient to see a healthcare provider who can deliver needed services in a period of time reasonable for their needs. (27) Access may be objective (e.g., the ability of a patients to schedule an appointment for a service within 30 days), or subjective (e.g., the ability of a patient to schedule an appointment in an amount of time that meets their preferences). This outcome represents how and the extent to which use of VHA virtual care technologies impacts patient perceptions of access to VHA services, as well as patient perceptions of their access to virtual care technologies themselves.	Self-report; Administrative data
**Perceived timeliness of services**	The timeliness of healthcare services refers to a healthcare system's ability to deliver services that a patient needs in a reasonable amount of time. (28) Much like access, timeliness can be subjective or objective. This outcome represents how and the extent to which use of VHA virtual care technologies impacts patient perceptions of the timeliness of VHA services.	Self-report; Administrative data

**Perceived quality and safety of care**	Quality and safety of healthcare encompasses the provision of effective care and reduction of errors in healthcare processes and practices, (29) which healthcare institutions may try to bolster through the integration of virtual care technologies into service delivery. (30) As with access and timeliness, healthcare quality and safety can be measured both objectively and subjectively. This outcome represents how and the extent to which use of VHA virtual care technologies impacts patient perceptions of the quality and safety of VHA services.	Self-report; Administrative data
**Perceived burden of care**	Burden of care encompasses tangible (e.g., physical, financial) and psychosocial (e.g., emotional, social) impacts of healthcare. (31) Some examples could include, but are not limited to, time spent in an appointment or time spent traveling to a VHA facility. The integration of virtual care technologies into service delivery may impact both objective and subjective burdens related to receipt of healthcare services. This outcome represents how and the extent to which use of VHA virtual care technologies impacts perceptions of healthcare burden among patients and their informal caregivers.	Self-report

Virtual Care Technology Access and Use Outcomes, Descriptions, and Possible Data Sources

Outcomes	Description	Possible Data Source(s)
**Access to devices and connectivity**	Access to technology – that is, device ownership and access to sufficient connectivity – is often discussed in the context of the "Digital Divide." (32) In order for virtual care technologies to be effectively integrated into care, stakeholders must have access to them. This outcome represents the extent to which stakeholders can obtain the connectivity (e.g., Wi-Fi, cellular data) and devices (e.g., mobile phones, tablets, PCs) necessary to use virtual care technologies.	Self-report; Administrative data
**Stakeholder awareness of virtual care technologies**	In addition to access, in order for an individual to access and use virtual care technologies, they must be aware of these technologies and their functionality. That is, awareness of a virtual care technology is a prerequisite to its use. This outcome represents the extent to which stakeholders are informed about virtual care	Self-report

	technologies, including their availability, features, and potential relevance to their own needs.	
**Stakeholder digital literacy**	Digital literacy has been defined as "the ability to use information and communication technologies to find, evaluate, create, and communicate information, requiring both cognitive and technical skills." (33) Digital literacy is relevant to all stakeholders and the use of all technologies. Inadequate digital literacy will hamper successful engagement with virtual care technologies. This outcome represents the extent of stakeholder digital literacy across VHA, including patient, their informal caregivers, and VHA care team member confidence to use virtual care technologies, as well as the provision of training and training opportunities for digital literacy.	Self-report
**Provision of virtual care options for all stakeholders**	Meeting patients where they are is a foundational concept in delivering patient-centered, Whole Health care. (4) To that end, it is imperative that VHA develops and provides a range of virtual care technologies that patients and informal caregivers can use based on their needs, preferences, and technology ownership/skills, but also recognize that for some, virtual care technologies may not be the most preferable option. Similarly, VHA care team members and staff may have different preferences for how and when to use virtual care technologies as part of their work. This outcome represents the extent to which VHA has developed and made available virtual care technologies (or alternatives) that align with stakeholder needs and preferences and meet them where they are.	Self-report
**Interactions with virtual care technologies and related data**	There are varying ways in which – and degrees to which – stakeholders could interact with the VHA virtual care technologies available to them, as well as their related data. This outcome represents how and the extent to which stakeholders use available VHA virtual care technologies and their related data.	Self-report; Administrative data; Activity log data
**Usability**	The usability of any system is a function of how well it accommodates user needs and their context. (34) This outcome represents how and the extent to which VHA virtual care technologies accommodate stakeholder needs and contexts.	Self-report; Activity log data
**Usefulness**	Usefulness refers to the degree to which a stakeholder believes that using a particular system would enhance or support the tasks they need to	Self-report

	perform. (35) This outcome represents how and the extent to which stakeholders find VHA virtual care technologies to be useful.	
**Acceptability**	Acceptability has been defined as the extent to which stakeholders find a service or product to be satisfactory, agreeable, and/or palatable. (36) This outcome represents the extent to which stakeholders find VHA virtual care technologies to be acceptable.	Self-report
**Feasibility**	Feasibility has been defined as how successfully a service or product can be used within a specific context. (37) This outcome represents the extent to which stakeholders find use of VHA virtual care technologies to be feasible.	Self-report; Administrative data; Activity log data
**Adoption**	Adoption refers to an individual's initial use of an innovation. (36) This outcome represents the extent to which stakeholders adopt various VHA virtual care technologies.	Self-report; Administrative data; Activity log data
**Sustained use**	Sustained use refers to the continued use of a service, product, or innovation after adoption. For some virtual care technologies, sustained use is needed for a stakeholder to fully realize intended benefits and what constitutes sustained use may look different for different technologies. This outcome represents the extent to which stakeholders sustain use of various VHA virtual care technologies over time.	Self-report; Administrative data; Activity log data
**Satisfaction with virtual care technologies**	"Customer satisfaction is defined as a measurement that determines how happy customers are with a company's products, services, and capabilities." (38) This outcome represents the extent to which stakeholders are satisfied with the virtual care technologies available to them.	Self-report
**Organizational readiness**	According to prominent theorists, (39) organizational readiness for change is a multi-level construct that can be present at the level of the individual, group, department, or broader organization. It is also multi-faceted, and encompasses the commitment to change among organizational members and their efficacy to implement organizational change. (40-41) This outcome represents the extent to which different organizational levels within VHA are ready to implement virtual care technologies.	Self-report; Administrative data
**TechQuity**	TechQuity has been defined as "the strategic development and deployment of technology in	Self-report;

	health care and health to achieve health equity." (42) Increasingly, there is recognition that how virtual care technologies are developed and deployed can impact the extent to which individuals can realize their best health and health outcomes. These impacts can be both intended and unintended, and be both positive and negative, thus emphasizing the importance of virtual care developers and implementers to embrace the concept of TechQuity. Recently, the concept of TechQuity has been introduced in relation to VHA and the patient population, recognizing the healthcare system's commitment to achieving equitable care and the diverse needs and barriers faced by members of the patient population. (43) This outcome represents how and the extent to which virtual care technologies help or hinder health equity.	Administrative data

Access to Care Outcomes, Descriptions, and Possible Data Sources

Outcomes	Description	Possible Data Source(s)
**Access to services (actual)**	Access to care refers to the ability of a patient to see a healthcare provider who can deliver needed services in a period of time reasonable for their needs. (27) Patient access to VHA healthcare services has been an important recent focus of VHA, with legislation like the MISSION Act expanding patient eligibility to receive care from community providers reimbursed by VHA. (44) This outcome represents how and the extent to which use of virtual care technologies impacts patient ability to receive needed healthcare services in an appropriate timeframe. Access to services (actual) is synergistic with Timeliness of services (actual).	Self-report; Administrative data
**Timeliness of services (actual)**	Timeliness of healthcare services refers to the ability of a healthcare system to deliver healthcare services that a patient needs, once this need for services becomes known. (28) Use of virtual care technologies may impact the timeliness of VHA services. This outcome represents how and the extent to which use of virtual care technologies impacts the speed at which patients receive the VHA healthcare services they want/need. Timeliness of	Self-report; Administrative data

	services (actual) is synergistic with Access to services (actual).	
**Travel time**	In the context of healthcare, travel time refers to the time a patient spends traveling to and from a healthcare facility. (45) The use of virtual care technologies may reduce the overall time patients spend traveling to a VHA facility, perhaps because some of their needs can be addressed remotely using technology. This outcome represents how and the extent to which integration of virtual care technologies impacts the amount of time patients and their informal caregivers spend traveling to and from VHA healthcare facilities.	Self-report; Administrative data; GPS/device data
**Appointment wait times**	Appointment wait times represent the amount of time that elapses between a patient's requests to schedule an appointment and when they are able to have that appointment. Additional metrics of appointment wait times, for example, for specialty care services, include the time that elapses from when an appointment consult is created and when that consult is approved, the time that elapses from when an appointment consult is approved and when the appointment corresponding to that consult is scheduled, and the time that elapses from when an appointment consult is approved and when the patient is able to have the corresponding appointment. (46) This outcome represents how and the extent to which the integration of virtual care technologies into VHA healthcare services impacts how long patients must wait to receive needed services.	Self-report; Administrative data

Utilization of Care Outcomes, Descriptions, and Possible Data Sources

Outcomes	Description	Possible Data Source(s)
**Utilization rates**	Certain healthcare services (e.g., inpatient stays, emergency department visits) are particularly expensive and comprise a substantive portion of costs incurred by healthcare systems. (47) The avoidance of such service utilization among patients, in cases where it is in fact avoidable, could result in substantive cost savings to VHA. It is also possible that some technologies will increase healthcare utilization because they engage	Self-report; Administrative data

	patients to address unmet healthcare needs. This outcome represents how and the extent to which use of virtual care technologies impact utilization of services – particularly, high-cost, avoidable services – among patients, and the resulting impacts on costs to the VHA healthcare system.	
**Supportive services via virtual care**	Virtual care technologies can be used to support a variety of care processes and interactions, including primary care services, as well as specialty care services (e.g., physical therapy). This outcome represents how and the extent to which virtual care technologies are leveraged to deliver or enhance VHA supportive services.	Self-report; Administrative data
**Remote management of patients/patient panels**	Remote patient monitoring entails healthcare team members monitoring symptoms of acute or chronic conditions (e.g., blood pressure, weight, blood sugar) from their patients while the patients are at home or otherwise not at a healthcare facility. (48) This outcome represents how and the extent to which VHA care team members use virtual care technologies to support remote monitoring of individual patients or of their patient panels.	Self-report; Administrative data; Activity log data
**Demand for services**	Demand for healthcare services refers to the relationship between care team member time and appointment volume. (49) Use of virtual care technologies may drive demand for services either up or down. This outcome represents how and the extent to which integration of virtual care technologies into practice impacts demand for VHA services.	Self-report; Administrative data

Quality of Care Outcomes, Descriptions, and Possible Data Sources

Outcomes	Description	Possible Data Source(s)
**Medication prescribing quality**	Prescribing medications is complex, particularly when a patient is faced with multiple health problems and comorbid conditions. Prominent entities including the World Health Organization have argued that prescribing medications should be viewed as a multi-step process, and other researchers have since expanded upon those ideas. (50-51) Efforts to measure prescribing quality have sometimes been criticized for their limited perspective, although some markers of prescribing	Self-report; Administrative data

	quality have been described in the literature, including the Beers Criteria, drug dosing, drug-drug interactions, drug-disease interactions, and the use of high-risk drugs. (52) Some specific measures of prescribing quality have also been articulated. These include: "1. taking any medication from the drugs-to-avoid criteria of Beers et al, 2. taking any medication with a score of 3 or more on the Medication Appropriateness Index (MAI), and/or 3. taking 9 or more medications (polypharmacy)." (53) This outcome represents how and the extent to which use of virtual care technologies impacts medication prescribing quality among VHA healthcare team members.	
**Meeting performance measures**	Performance measurement has been defined as the assessment of specific outcomes for insight on program effectiveness and/or efficiency. (54) The VHA regularly assesses VHA Medical Center performance using their Strategic Analytics for Improvement and Learning Value Model (SAIL), which encompasses measures including but not limited to mortality, patient satisfaction, and medical complications. (55) The integration of virtual care technologies into service delivery has the potential to help or hinder VHA care team member ability to meet performance measures. This outcome represents how and the extent to which use of virtual care technologies impacts healthcare team member ability to meet established performance metrics.	Self-report; Administrative data
**Efficiency**	In the context of healthcare, efficiency has been defined as "whether healthcare resources are being used to get the best value for money." (56) This outcome represents the extent to which use of virtual care technologies impacts efficiency of VHA services.	Self-report; Administrative data
**Care coordination**	Care coordination entails sharing a patient's health- and healthcare-related information among all individuals who have involvement with their healthcare to optimize safety and impacts of treatment. (57-59) This outcome represents how and the extent to which the use of virtual care technologies impacts patient care coordination.	Self-report; Administrative data
**Fragmentation of care**	In juxtaposition to care coordination, fragmentation of care occurs when multiple care providers make treatment plans/recommendations for a patient but do not communicate with one another regarding the interplay of these plans/recommendations. (60) This outcome represents how and the extent to which the use of virtual care technologies impacts fragmentation of patient healthcare.	Self-report; Administrative data

**Care transitions**	Care transitions refers to a patient being moved from one care setting or context to another, (61) for example, from hospital to home. Such transitions can be complicated and exacerbate potential for adverse events. (57) This outcome represents how and the extent to which the use of virtual care technologies impacts safe and effective care transitions for patients.	Self-report
**Readmissions**	Preventing readmissions following a hospital discharge is a high priority and marker of improved healthcare quality as well as a means to lower healthcare costs. (62) Virtual care technologies may support patient transitions from hospital to home, communication with care team members, and self-management, which may in turn prevent avoidable readmissions. This outcome represents how and the extent to which the use of virtual care technologies impacts patient readmissions following an inpatient stay.	Self-report; Administrative data
**Community tenure**	Akin to preventing readmissions, healthcare organizations may assess an individual's community tenure, that is, the length of time an individual is able to live in a community setting (as opposed to an inpatient or other long-term care setting). This outcome represents how and the extent to which the use of virtual care technologies impacts patient ability to reside in the community.	Self-report; Administrative data
**Patient Safety**	The World Health Organization has defined patient safety as "the absence of preventable harm to a patient and reduction of risk of unnecessary harm associated with health care to an acceptable minimum." (63) Across care contexts, safety concerns can emerge from a variety of sources, including but not limited to healthcare procedures, prescription medications or other treatments, the potential for infections, and even poor information management. (63) These sources of safety concerns can be complicated by a range of factors, including organizational issues, technologies, stakeholder behaviors, and broader influences (e.g., national policies) that extend beyond a healthcare setting itself. (63) This outcome represents how and the extent to which the use of virtual care technologies impacts the safety of VHA healthcare services delivered to patients.	Self-report; Administrative data
**Receipt of preventive care**	Preventive care encompasses actions intended to detect potential health problems before they become serious and thus promotes overall health and well-being. (64) Common preventive care services include but are not limited to screening for different conditions, receiving suggested immunizations, and seeking guidance on	Self-report; Administrative data

	lifestyle and dietary choices. (65) This outcome represents how and the extent to which the use of virtual care technologies impacts the receipt of preventive care services among patients.	
**Extent of evidence-based treatment**	Evidence-based practice is the provision of healthcare services based on the best evidence available at the time coupled with a healthcare provider’s clinical experience and expertise. (66) This outcome represents how and the extent to which the integration of virtual care technologies into practice impacts the ability of VHA care team members to provide, and patients to engage with, evidence-based treatments.	Self-report; Administrative data
**Can we provide equivalent or better quality services via technology**	As the integration of technology into healthcare becomes more commonplace, the impacts of technology on healthcare quality relative to in-person services must be examined. At present, research on such impacts shows promising results in certain contexts of care, but more research is needed. (67) This outcome represents how and the extent to which healthcare services delivered via technology impacts their quality, relative to the delivery of those services offered independent of technology.	Self-report; Administrative data

Clinical Indices Outcomes, Descriptions, and Possible Data Sources

Outcomes	Description	Possible Data Source(s)
**Morbidity and mortality**	“Morbidity is the state of being symptomatic or unhealthy [in the context of a particular] disease or condition. Mortality is related to the number of deaths caused by the [disease or condition] under investigation.” (68) This outcome represents how and the extent to which use of virtual care technologies impacts morbidity and mortality among patients who receive VHA healthcare services.	Self-report; Administrative data; PGHD
**Physiological markers**	Physiological biomarkers have been defined as “a characteristic that is objectively measured and evaluated as an indicator of normal biological processes, pathogenic processes, or pharmacologic responses to a therapeutic intervention.” (69) Examples of such markers include but are not limited to: blood pressure, hemoglobin A1c, oxygen, cholesterol, body weight, and heart rate. This outcome represents how and the extent to which virtual care technologies are used to collect data regarding physiological biomarkers and to	Self-report; Administrative data; PGHD

	communicate those data to healthcare team members, or in an effort to improve physiological biomarkers among patients.	
**Mental health status**	Mental health status can include information regarding a patient's behavioral functioning, cognitive functioning, and symptoms (e.g., depression, anxiety). (70) As with physiological biomarkers, virtual care technologies can help VHA care team members collect information on patient mental health status, or can be used as part of intervention(s) intended to support patient mental health. This outcome represents how and the extent to which virtual care technologies are used to collect data regarding patient mental health status and to communicate those data to healthcare team members, or in an effort to improve mental health status among patients.	Self-report; Administrative data; PGHD
**Condition/disease burden**	The concept of disease burden can encompass many related but distinct constructs, including but not limited to morbidity and mortality, costs (including healthcare-related costs and indirect costs such as productivity loss), and negative social and emotional impacts. (71) This outcome represents how and the extent to which the integration of virtual care technologies into practice impact disease burden among patients.	Self-report; Administrative data; PGHD
**Medication regimen complexity**	Medication regimen complexity refers to the number of medications that an individual is taking, as well as the frequency and type of dosing associated with those medications. (72) This outcome represents how and the extent to which use of virtual care technologies impact patient medication regimen complexity and medication management.	Self-report; Administrative data

Function Outcomes, Descriptions, and Possible Data Sources

Outcomes	Description	Possible Data Source(s)
**Functional status**	Functional status can be thought of as "the degree to which an individual can perform chosen roles without limitation in three key domains: physical, social, and psychocognitive." (73) Functional status concerns how an individual's daily activities and social interactions are impacted by health condition(s). This outcome represents how and the extent to which use of virtual care technologies impacts patient functional status.	Self-report; Administrative data

**Quality of life**	Health-related quality of life has been defined as an individual's perception of their physiological and psychosocial health, as well as an assessment of related issues including health conditions, social support, and functioning. (74) This outcome represents how and the extent to which virtual care technologies are used to collect data regarding patient quality of life and communicate those data to healthcare team members, or in an effort to improve quality of life among patients.	Self-report; Administrative data

Health Promotion and Self-Management Outcomes, Descriptions, and Possible Data Sources

Outcomes	Description	Possible Data Source(s)
**Adherence with medications or treatment plans**	In the context of health, adherence has been defined as "the extent to which a person's behavior – taking medication, following a diet, and/or executing lifestyle changes – corresponds with agreed recommendations from a health care provider." (75) This outcome represents how and the extent to which use of virtual care technologies impacts patient adherence to their care, including following their prescription medications and broader treatment plans.	Self-report; Administrative data; PGHD
**Health behavior**	Health behaviors refer to those behaviors performed by an individual which could impact their health, including but not limited to physical activity, diet, smoking, and sleep. (76) This outcome represents how and the extent to which use of virtual care technologies impacts patient health behaviors.	Self-report; Administrative data; PGHD
**Facilitating self-management**	Self-management encompasses an individual's engagement in managing their health and/or health conditions. (57) Virtual care technologies may support self-management among patients. This outcome represents how and the extent to which use of virtual care technologies impacts patient management of their health/health conditions.	Self-report
**Management of personal health information**	"Personal health information management refers to activities that support consumers' access, integration, organization, and use of their personal health information." (77) This outcome represents how and the extent to which use of virtual care technologies impact personal health information management among patients.	Self-report; Administrative data

**Health literacy and health-related knowledge**	Health literacy refers to one's ability to obtain, process, and make use of information in the context of decisions about one's health and healthcare, or the health and healthcare of others. (78) Possessing knowledge about one's health or the health of others is an important component of health literacy. This outcome represents how and the extent to which use of virtual care technologies impacts patient health and healthcare-related knowledge.	Self-report
**Self-efficacy**	As noted in our discussion of healthcare team member outcomes, self-efficacy refers to the perceptions of an individual regarding their ability to do something (e.g., perform a task, engage in a behavior). (13) In the context of healthcare, virtual care technologies may impact patient self-efficacy related to managing their health and healthcare. This outcome represents how and the extent to which use of virtual care technologies impacts patient confidence to manage their health/healthcare.	Self-report
**Activation**	Patient activation – that is, the understanding and ability of an individual to manage their health and health care – has been associated with improved health outcomes and experiences of care. (79) This outcome represents how and the extent to which use of virtual care technologies impacts patient activation.	Self-report
**Social support (actual, perceived)**	Social support refers to the receipt of help from others to address needs that an individual may be facing. (80) Conceptual analysis has identified defining attributes or types of social support that individuals can receive. Among the most common include: emotional, instrumental, informational, and appraisal. (81) This outcome represents how and the extent to which use of virtual care technologies impacts social support received or perceived by patients and their informal caregivers.	Self-report
**Social isolation**	Considerable research has highlighted the strong association between social connectedness and an individual's mental and physical health and trajectories of healthy aging. Having more and stronger connections to others is increasingly recognized as a core determinant of most health outcomes, emphasizing the importance of identifying and supporting those who may face feelings of loneliness. (82-84) This outcome represents how and the extent to which use of	Self-report

	virtual care technologies impacts social isolation among patients.	
**Housing stability**	Homelessness has been recognized as a pressing problem in the patient population; however, other challenges like having an inconsistent place to shelter or a shelter that is of substandard quality present an equally compelling problem for many patients. Housing stability encompasses access to and the continued availability of housing that is safe, comfortable, affordable, and sustainable. This outcome represents how and the extent to which use of virtual care technologies impacts the experience of housing stability among patients. (85)	Self-report; Administrative data
**Stress**	Stress has been defined as "the body's response to physical, mental, or emotional pressure" and experiences of stress have been linked with poor physical and mental health outcomes. (86) This outcome represents how and the extent to which use of virtual care technologies impact experiences and management of stress among patients and their informal caregivers.	Self-report
**Goal setting, progress, achievement**	Goal setting refers to the identification of a wanted outcome and the steps that need to be taken to achieve that outcome. (87) In order for health-related goals to be most attainable, it is important that they possess the characteristics of a SMART goal, that is, that they are specific, measurable, attainable, realistic, and trackable. (88) This outcome represents how and the extent to which use of virtual care technologies impact patient health-related goal setting, goal progress, and goal achievement.	Self-report
**Caregiver burden**	Caregiver burden refers to the physical, psychological, and social strain that results from informal caregiving – the provision of assistance to a patient by a family member, friend, or other loved one, without expectation of compensation or other remuneration. (89) The integration of virtual care technologies into patient care, as well as use of virtual care technologies by informal caregivers themselves, may influence experiences of burden. This outcome represents how and the extent to which use of virtual care technologies impact feelings of burden among a patient's informal caregivers.	Self-report

**Psychosocial benefits of caregiving**	Although serving in the role of caregiver to a patient can present challenges, there have also been documented benefits from serving in the caregiving role. Many caregivers have reported positive experiences, including but not limited to feelings of meaning and purpose, satisfaction knowing a loved one is receiving quality care, and a sense of giving back and personal growth. (90) The recognition of these benefits may help caregivers cope with the burdens and stress of the role. Caregivers may also experience positive emotions like a sense of satisfaction and growth, while simultaneously feeling distressed. (91-92) This outcome represents how and the extent to which use of virtual care technologies impact feelings of psychosocial benefit among a patient's informal caregivers.	Self-report
**Patient sexual and reproductive health behaviors**	Sexual and reproductive health behaviors include a spectrum of different behaviors ranging from sexual function and disease prevention to the use of contraceptives, engaging in routine disease screening, and sexual adjustments in accordance with aging, illness, and disability. (93) Taken together, sexual and reproductive health behaviors help to avert health distress and enhance sexual and reproductive function. (93) This outcome represents how and the extent to which use of virtual care technologies impact sexual and reproductive behavior among patients.	Self-report; Administrative data
**Patient and caregiver well-being**	Recently, VHA implemented a definition of well-being that encompasses the extent to which patients are thriving in daily life, including having acceptable living conditions and positive emotions about their overall life (e.g., purpose and satisfaction) as well as more specific life domains (e.g., social, health, financial, etc.). (94) Because well-being is personal and subjective, it is typically measured through self-report approaches, and at present, there are a variety of well-being instruments available to measure the concept, including VHA's recently developed Well-Being Signs Measure. (95) This outcome represents how and the extent to which use of virtual care technologies impact well-being among patients and their informal caregivers.	Self-report; Administrative data
**Healthcare decision-making**	Seeking and receiving healthcare services is a complex undertaking, and patients are often faced with a need to determine how they will manage	Self-report

	their health and conditions, including deciding among treatments that may be available to them. Making such decisions can be challenging, and associated with feelings of uncertainty, stress, and confusion. (96) Virtual care technologies have the potential to impact the processes by which patients make decisions about their health and healthcare, possibly easing the burden in some cases or adding to the burden in others. This outcome represents how and the extent to which use of virtual care technologies impact healthcare decision-making among patients.	
**Patient interpersonal and relationship dynamics**	Interpersonal and relationship dynamics encompasses the interactions among individuals in a particular context and how they relate to one another. (97) These patterns of behavior and communication can span a continuum from healthy to unhealthy and be associated with a variety of emotions. This outcome represents how and the extent to which use of virtual care technologies impact the interpersonal and relationship dynamics that patients have with others in their lives.	Self-report

Costs to the Healthcare System Outcomes, Descriptions, and Possible Data Sources

Outcomes	Description	Possible Data Source(s)
**Community care**	Although VHA has long had a fee basis program, wherein VHA would cover the costs of community-based care for patients in specific situations, more recent legislation (e.g., the MISSION Act) further expanded the circumstances in which VHA would reimburse for community-based care. (44) Virtual care platforms, such as synchronous telehealth technology, may increase patient ability to engage with a VHA (rather than community-based) care team member, even in situations where that care could have been outsourced to the community, like when a patient lives far away from a VHA facility. This outcome represents how and the extent to which use of virtual care technologies impact the amount of money VHA spends on community care for patients.	Self-report; Administrative data
**Travel reimbursement**	For those patients who are eligible, VHA will reimburse them and their informal caregivers for expenses incurred as a result of traveling to and from healthcare encounters. Virtual care	Self-report; Administrative data

	technologies, such as telehealth platforms and secure messaging, may impact patient travel for care and in turn, VHA travel reimbursement. This outcome represents how and the extent to which use of virtual care technologies impact the amount of money VHA spends reimbursing patients and their informal caregivers for travel to and from care encounters.	
**Cost savings and avoidance**	Cost savings refers to actions that result in decreases in spending, while cost avoidance refers to actions that result in avoiding incurred future costs. (98) Virtual care technologies may impact the ability of VHA to save and avoid costs through various mechanisms (e.g., increased patient engagement and improved outcomes, which could lead to decreased downstream costs, increased productivity among care team members). This outcome represents how and the extent to which use of virtual care technologies impact the amount of money VHA currently spends, as well as the amount of money the healthcare system will spend in the future.	Self-report; Administrative data
**Total cost per patient**	In the context of healthcare, total cost per patient refers to the total expenditures a healthcare system incurs for a single patient in any given time frame. According to the IHI, the "total cost per member of the population per month is the desirable measure for per capita cost." (47) This outcome represents how and the extent to which use of virtual care technologies impact the amount of money VHA spends delivering care to any given patient in a specified amount of time.	Self-report; Administrative data
**Operational costs**	In the context of healthcare, operational costs include money spent on labor, as well as non-labor-related expenditures, related to inpatient and outpatient services, ancillary services, and overhead. (99) This outcome represents how and the extent to which use of virtual care technologies impact VHA operational costs.	Self-report; Administrative data
**Virtual care technology development cost**	Costs related to the development of various technologies differ, in part based on the complexity and requirements of the technology at hand. As VHA continues to support the development of new and cutting-edge virtual care technologies, the costs related to this development must be considered. This outcome represents how and the extent to	Self-report; Administrative data

	which the development of virtual care technologies impacts costs to the VHA healthcare system.	
**Virtual care technology maintenance cost**	Once a technology has been developed, the technology must be updated and/or maintained over time as operating systems, software, and hardware evolve. This outcome represents how and the extent to which the maintenance of virtual care technologies impacts costs to the VHA healthcare system.	Self-report; Administrative data
**Virtual care technology implementation cost**	The VHA OCC engages in ongoing initiatives intended to support the adoption and use of its virtual care technologies among patients and VHA care team members and staff; these implementation activities have associated costs. This outcome represents how and the extent to which the cost of implementing virtual care technologies impacts costs to the VHA healthcare system.	Self-report; Administrative data
**Cost effectiveness**	Cost effectiveness refers to the comparison of costs and associated impacts on health outcomes among different interventions. (100) In the context of virtual care technologies, costs can be compared for interventions that do and do not involve use of technologies, as well as different technology-based interventions. This outcome represents how and the extent to which use of virtual care technologies impact cost effectiveness for interventions offered by VHA.	Self-report; Administrative data

Costs to the Patient Outcomes, Descriptions, and Possible Data Sources

Outcomes	Description	Possible Data Source(s)
**Travel costs**	Patient travel costs related to healthcare encounters may be incurred from terminal milage, tolls, and/or parking fees; costs related to public transportation, ride shares/taxis, or flights; and meals and lodging (as needed). (101) Use of virtual care technologies may impact the costs that patients incur related to traveling to and from healthcare encounters. This outcome represents how and the extent to which use of virtual care technologies impact the amount of money that patients spend on travel-related expenses to receive healthcare services.	Self-report; Administrative data
**Copays and premiums**	Although some patients are exempt from having to pay copayments for VHA services, others incur costs related to copayments and premiums for medication and medical services. (102) This	Self-report; Administrative data

	outcome represents how and the extent to which use of virtual care technologies impacts the amount of money that patients spend on copayments and premiums for healthcare services.	
**Lost days of work**	Productivity loss – that is, the inability of an employee to present to work, or to be optimally productive while at work, due to health conditions and/or related symptoms, can be costly for employers. (103) In addition, productivity loss can be costly to patients themselves, for example, if they are working for an hourly wage (as opposed to a salaried position) and cannot work as many hours as they would like. This outcome represents how and the extent to which use of virtual care technologies impacts the amount of productivity loss that patients experience as a result of their health/healthcare.	Self-report
**Cost savings and avoidance**	As described above, cost savings refers to actions that result in decreases in spending, while cost avoidance refers to actions that result in avoiding incurred future costs. (98) Virtual care technologies may impact patient ability to save and avoid costs related to their health and healthcare. This outcome represents how and the extent to which use of virtual care technologies impacts the amount of money patients currently spend on health/healthcare-related expenses, as well as the amount of money they will spend on such expenses in the future.	Self-report; Administrative data
**Cost effectiveness**	As described above, cost effectiveness refers to the comparison of costs and associated impacts on health outcomes among different interventions, (100) for example, among interventions that do and do not involve use of technologies, or for different technology-based interventions. This outcome represents how and the extent to which use of virtual care technologies impacts cost effectiveness for VHA healthcare services for patients and their informal caregivers.	Self-report; Administrative data

4 References

1. Encyclopædia Britannica. Convenience. In: The Britannica dictionary [Internet]. [cited 2025 Jul 1]. Available from: https://www.britannica.com/dictionary/convenience

2. James J. Patient engagement [Internet]. Hibbard J, Agres T, Lott R, Dentzer S, editors. Health Affairs. 2013 Feb [cited 2025 Jul 1]. Available from: https://www.healthaffairs.org/content/briefs/patient-engagement

3. Higgins T, Larson E, Schnall R. Unraveling the meaning of patient engagement: a concept analysis. Patient Educ Couns. 2017 Jan 1;100(1):30-6. doi: 10.1016/j.pec.2016.09.002
4. U.S. Department of Veterans Affairs. Whole health [Internet]. VA.gov. [updated 2025 June 27; cited 2025 Jul 1]. Available from: https://www.va.gov/wholehealth/
5. Centers for Medicare & Medicaid Services. Health care transactions basics [Internet]. CMS.gov. [cited 2025 Jul 1]. Available from: https://www.cms.gov/files/document/health-care-transactions-basics.pdf
6. Veterans In Communities. Veteran to veteran peer support [Internet]. Veterans In Communities. [cited 2025 Jul 1]. Available from: https://www.veteransincommunities.org/veteran-to-veteran-peer-support.html
7. Heath S. What Are HCAHPS scores, why are they important to patient satisfaction? [Internet]. TechTarget. 2022 [cited 2025 Jul 1]. Available from: https://www.techtarget.com/patientengagement/feature/What-Are-HCAHPS-Scores-Why-Are-They-Important-to-Patient-Satisfaction
8. Fishbein D, Nambiar S, McKenzie K, Mayorga M, Miller K, Tran K, Schubel L, Agor J, Kim T, Capan M. Objective measures of workload in healthcare: a narrative review. Int J Health Care Qual Assur. 2019 Dec 30;33(1):1-17. doi:10.1108/ijhcqa-12-2018-0288
9. Agency for Healthcare Research and Quality. What is workflow? [Internet]. Ahrq.gov. [cited 2025 Jul 1]. Available from: https://digital.ahrq.gov/health-it-tools-and-resources/evaluation-resources/workflow-assessment-health-it-toolkit/workflow
10. Iyengar MS, Rogith D, Florez-Arango JF. Measuring workload demand of informatics systems with the clinical case demand index. In: AMIA Annu Symp Proc [Internet]. 2017 [cited 2025 Jul 1]. p. 985–93. Available from: https://pmc.ncbi.nlm.nih.gov/articles/PMC5977563/
11. Maslach C, Jackson SE. The measurement of experienced burnout. J Organ Behav. 1981 Apr;2(2):99-113. doi:10.1002/job.4030020205
12. Spector PE. Job satisfaction: application, assessment, causes, and consequences [Internet]. Sagepub.com. Thousand Oaks, CA: SAGE Publications, Inc.; 2012 [cited 2025 Jul 1]. Available from: https://sk.sagepub.com/book/mono/job-satisfaction/toc
13. Bandura A. Self-efficacy. In: Ramachaudran VS, editor. Encyclopedia of human behavior [Internet]. New York: Academic Press; 1994 [cited 2025 Jul 28]. Available from: https://happyheartfamilies.citymax.com/f/Self_Efficacy.pdf
14. NHS Education for Scotland. Clinical decision making [Internet]. Effective Practitioner. [cited 2025 Jul 1]. Available from: https://www.effectivepractitioner.nes.scot.nhs.uk/media/254840/clinical%20decision%20making.pdf
15. Elwyn G, Frosch D, Thomson R, Joseph-Williams N, Lloyd A, Kinnersley P, Cording E, Tomson D, Dodd C, Rollnick S, Edwards A, Barry M. Shared decision making: a model for clinical practice. J Gen Intern Med. 2012 May 23;27(10):1361-1367. doi:10.1007/s11606-012-2077-6

16. U.S. Department of Veterans Affairs. What is whole health? Published July 3, 2025. Accessed July 30, 2025. https://www.va.gov/wholehealth/
17. Spink LM. Six steps to patient rapport. AD Nurse. 1987;2(2):21-3.
18. Community Preventive Services Task Force. Health communication and health information technology [Internet]. The Community Guide. [updated 2025 Jan 7;cited 2025 Jul 1]. Available from: https://www.thecommunityguide.org/topics/health-communication-and-health-information-technology.html
19. Sanson-Fisher RW, Campbell EM, Redman S, Hennrikus DJ. Patient-provider interactions and patient outcomes. Diabetes Educ. 1989 Apr;15(2):134-138. doi:10.1177/014572178901500209
20. Terrell Hanna K. What is brand recognition? [Internet]. TechTarget. 2023 [cited 2025 Jul 1]. Available from: https://www.techtarget.com/searchcustomerexperience/definition/brand-recognition
21. U.S. Department of Veterans Affairs. Tier 1 graphic standards: foundation for brand maintenance and evolution [Internet]. VA.gov. 2012 [cited 2025 Jul 1]. Available from: https://www.va.gov/opa/publications/graphicstandards/va_graphicstandardsguide_508_0113.pdf
22. Cambridge University Press & Assessment. Trust. In: Cambridge Dictionary [Internet]. [cited 2025 Jul 1]. Available from: https://dictionary.cambridge.org/us/dictionary/english/trust
23. U.S. Department of Veterans Affairs. Veteran trust in VA [Internet]. VA.gov. [updated 2025 May 7; cited 2025 Jul 1]. Available from: https://www.va.gov/initiatives/veteran-trust-in-va/
24. U.S. Department of Veterans Affairs. Community care. Published September 10, 2014. Accessed August 19, 2022. https://www.va.gov/COMMUNITYCARE/
25. U.S. Department of Veterans Affairs. Hiring programs and incentives [Internet]. VA.gov. [cited 2025 Jul 2]. Available from: https://www.va.gov/jobs/hiring_programs.asp
26. VA Careers. Our PACT with you: How VA is improving recruitment, retention [Internet]. VA News. 2022 [cited 2025 Jul 2]. Available from: https://news.va.gov/105744/our-pact-with-you-how-va-is-improving-recruitment-retention/
27. Health Affairs. Access to care [Internet]. www.healthaffairs.org. [cited 2025 Jun 27]. Available from: https://www.healthaffairs.org/topic/2233
28. Office of Disease Prevention and Health Promotion. Access to health services [Internet]. Healthy People. [cited 2025 Jul 3]. Available from: https://wayback.archive-it.org/5774/20220413202227/https://www.healthypeople.gov/2020/topics-objectives/topic/Access-to-Health-Services
29. Institute of Medicine. Patient safety: achieving a new standard for care [Internet]. Aspden P, Corrigan JM, Wolcott J, Erickson SM, editors. Washington, D.C.: The National Academies Press; 2004 [cited 2019 May 10]. Available from: https://nap.nationalacademies.org/catalog/10863/patient-safety-achieving-a-new-standard-for-care
30. American Hospital Association. Improving patient safety and health care quality through health information technology [Internet]. Washington, D.C.: American Hospital Association;

2018 Jul [cited 2025 Jun 29]. Available from: https://www.aha.org/system/files/2018-07/18-07-trendwatch-issue-brief3-patient-safety-quality-health-it.pdf

31. O'Neill G, Ross MM. Burden of care: an important concept for nurses. Health Care Women Int. 1991;12(1):111-121. doi:10.1080/07399339109515931

32. Appold K. Closing the digital divide: ensuring patients have access to healthcare technology is a priority. ENTtoday [Internet]. 2021 Aug 21 [cited 2025 Jul 3]; Available from: https://www.enttoday.org/article/closing-the-digital-divide-ensuring-patients-have-access-to-healthcare-technology-is-a-priority/?singlepage=1

33. American Library Association. Digital literacy [Internet]. ALA Literacy Clearinghouse. [cited 2025 Jul 8]. Available from: https://literacy.ala.org/digital-literacy/

34. Interaction Design Foundation. Usability [Internet]. Interaction Design Foundation. 2016 [cited 2025 Jul 8]. Available from: https://www.interaction-design.org/literature/topics/usability

35. Davis FD. Perceived usefulness, perceived ease of use, and user acceptance of information technology. MIS Quarterly. 1989 Sep;13(3):319-340. doi:10.2307/249008

36. Proctor E, Silmere H, Raghavan R, Hovmand P, Aarons G, Bunger A, Griffey R, Hensley M. Outcomes for implementation research: conceptual distinctions, measurement challenges, and research agenda. Adm Policy Ment Health. 2011;38(2):65-76. doi:10.1007/s10488-010-0319-7

37. Karsh BT. Beyond usability: designing effective technology implementation systems to promote patient safety. Qual Saf Health Care. 2004 Oct 1;13(5):388-394. doi:10.1136/qhc.13.5.388

38. American Society for Quality. What is customer satisfaction? [Internet]. ASQ. [cited 2025 Jul 8]. Available from: https://asq.org/quality-resources/customer-satisfaction

39. Weiner BJ. A theory of organizational readiness for change. Implement Sci. 2009 Oct 19;4:67. doi:10.1186/1748-5908-4-67

40. Weiner BJ, Amick H, Lee SY. Conceptualization and measurement of organizational readiness for change: a review of the literature in health services research and other fields. Med Care Res Rev. 2008 Feb 14;65(4):379-436. doi:10.1177/1077558708317802

41. Weiner BJ, Lewis MA, Linnan LA. Using organization theory to understand the determinants of effective implementation of worksite health promotion programs. Health Educ Res. 2009 Apr;24(2):292-305. doi:10.1093/her/cyn019

42. Rhee K, Dankwa-Mullan I, Brennan V, Clark C. What is TechQuity? J Health Care Poor Underserved. 2021 May;32(2):xiii–xviii. doi:10.1353/hpu.2021.0045

43. Walsh C, Sullivan C, Bosworth HB, Wilson S, Gierisch JM, Goodwin KB, Mccant F, Hoenig H, Heyworth L, Zulman DM, Turvey C, Moy E, Lewinski AA. Incorporating TechQuity in virtual care within the Veterans Health Administration: identifying future research and operations priorities. J Gen Intern Med. 2023 Jul;38(9):2130-2138. doi:10.1007/s11606-023-08029-2.

44. Congressional Budget Office. The Veterans Community Care Program: background and early effects [Internet]. Congressional Budget Office. 2021 Oct [cited 2025 Jul 9]. Available from: https://www.cbo.gov/publication/57583

45. Weiss DJ, Nelson A, Vargas-Ruiz CA, Gligorić K, Bavadekar S, Gabrilovich E, Bertozzi-Villa A, Rozier J, Gibson HS, Shekel T, Kamath C, Lieber A, Schulman K, Shao Y, Qarkaxhija V, Nandi AK, Keddie SH, Rumisha S, Amratia P, Arambepola R, Chestnutt EG, Millar JJ, Symons TL, Cameron E, Battle KE, Bhatt S, Gething PW. Global maps of travel time to healthcare facilities. Nat Med. 2020 Sep 28;26(12):1835-1838. doi:10.1038/s41591-020-1059-1

46. Griffith KN, Ndugga NJ, Pizer SD. Appointment wait times for specialty care in Veterans Health Administration facilities vs community medical centers. JAMA Netw Open. 2020 Aug 26;3(8):e2014313. doi:10.1001/jamanetworkopen.2020.14313

47. Stiefel M, Nolan K. A guide to measuring the triple aim: population health, experience of care, and per capita cost [Internet]. Institute for Healthcare Improvement. Cambridge, MA: IHI Innovation Series white paper; 2012 [cited 2025 Jun 27]. Available from: https://www.ihi.org/resources/white-papers/guide-measuring-triple-aim-population-health-experience-care-and-capita-cost#downloads

48. Health Resources & Services Administration. Telehealth and remote patient monitoring [Internet]. telehealth.hhs.gov. [updated 2024 Aug 28; cited 2025 Jul 9]. Available from: https://telehealth.hhs.gov/providers/preparing-patients-for-telehealth/telehealth-and-remote-patient-monitoring/

49. Turley S, Hirschman M, McMillen S. Balancing the supply and demand of patient access [Internet]. Medical Group Management Association. 2020 [cited 2025 Jul 9]. Available from: https://www.mgma.com/articles/balancing-the-supply-and-demand-of-patient-access

50. Pollock M, Bazaldua OV, Dobbie AE. Appropriate prescribing of medications: an eight-step approach. Am Fam Physician. 2007 Jan 15;75(2):231-236. Available from: https://www.aafp.org/pubs/afp/issues/2007/0115/p231.html

51. de Vries TPGM, Henning RH, Hogerzeil HV, Fresle DA. Guide to good prescribing [Internet]. [cited 2025 Jul 9]. Available from: https://iris.who.int/bitstream/handle/10665/59001/WHO_DAP_94.11.pdf

52. Steinman MA, Miao Y, Boscardin WJ, Komaiko KD, Schwartz JB. Prescribing quality in older veterans: a multifocal approach. J Gen Intern Med. 2014 Jul 8;29(10):1379-1386. doi:10.1007/s11606-014-2924-8

53. Steinman MA, Rosenthal GE, Landefeld CS, Bertenthal D, Sen S, Kaboli PJ. Conflicts and concordance between measures of medication prescribing quality. Med Care. 2007 Jan;45(1):95-99. doi:10.1097/01.mlr.0000241111.11991.62

54. Evaluation division, Bureau of Educational and Cultural Affairs. Performance measurement definitions [Internet]. [cited 2025 Jul 28]. Available from: https://eca.state.gov/files/bureau/performance_measurement_definitions.pdf

55. U.S. Department of Veterans Affairs. SAIL FY2021 hospital performance - all facilities [Internet]. Department of Veterans Affairs Open Data Portal. [updated 2022 Jan 10; cited 2025 Jul 10]. Available from: https://www.data.va.gov/dataset/SAIL-FY2021-Hospital-Performance-All-Facilities/y9x8-349i/about_data

56. Palmer S, Torgerson DJ. Economic notes: definitions of efficiency. BMJ. 1999 Apr 24;318(7191):1136. doi:10.1136/bmj.318.7191.1136

57. Hogan TP, Nazi KM, Luger TM, Amante DJ, Smith BM, Barker A, Shimada SL, Volkman JE, Garvin L, Simon SR, Houston TK. Technology-assisted patient access to clinical information: an evaluation framework for blue button. JMIR Res Protoc. 2014 Mar 27;3(1):e18. doi:10.2196/resprot.3290

58. Bodenheimer T. Coordinating care--a perilous journey through the health care system. N Engl J Med. 2008 Mar 6;358(10):1064-1071. doi:10.1056/NEJMhpr0706165

59. Agency for Healthcare Research and Quality. Care coordination [Internet]. Ahrq.gov. 2014 [updated 2024 Nov; cited 2025 Jul 10]. Available from: https://www.ahrq.gov/ncepcr/care/coordination.html

60. Elhauge E. The fragmentation of U.S. health care: causes and solutions [Internet]. New York: Oxford Academic; 2010 [cited 2025 Jul 10]. Available from: https://academic.oup.com/book/12404

61. Agency for Healthcare Research and Quality. Chartbook on care coordination: transitions of care [Internet]. Ahrq.gov. 2016 [updated 2018 Jun; cited 2025 Jul 10]. Available from: https://www.ahrq.gov/research/findings/nhqrdr/chartbooks/carecoordination/measure1.html

62. Agency for Healthcare Research and Quality. Hospital readmissions [Internet]. Ahrq.gov. [cited 2025 Jul 10]. Available from: https://www.ahrq.gov/topics/hospital-readmissions.html

63. World Health Organization. Patient Safety [Internet]. World Health Organization. 2023 [cited 2025 Jul 10]. Available from: https://www.who.int/news-room/fact-sheets/detail/patient-safety

64. Centers for Medicare & Medicaid Services. Prevention [Internet]. medicaid.gov. [cited 2025 Jul 29]. Available from: https://www.medicaid.gov/medicaid/benefits/prevention

65. U.S. Department of Health and Human Services. Preventive Care [Internet]. hhs.gov [cited 2025 Jul 29]. Available from: https://www.hhs.gov/healthcare/about-the-aca/preventive-care/index.html

66. Agency for Healthcare Research and Quality. Topic: evidence-based practice [Internet]. Ahrq.gov. [cited 2025 Jul 10]. Available from: https://www.ahrq.gov/topics/evidence-based-practice.html

67. California Health Benefits Review Program. Background brief: telehealth: current state of the evidence [Internet]. California Health Benefits Review Program. 2021 Feb [cited 2025 Jul 10]. Available from: https://www.chbrp.org/sites/default/files/Telehealth%20Background%20Brief-%20FINAL.pdf

68. Hernandez JBR, Kim PY. Epidemiology morbidity and mortality [Internet]. National Institutes of Health. Treasure Island, FL: StatPearls Publishing; [cited 2025 Jul 10]. Available from: https://www.ncbi.nlm.nih.gov/books/NBK547668/

69. Biomarkers Definitions Working Group. Biomarkers and surrogate endpoints: preferred definitions and conceptual framework. Clin Pharmacol Ther. 2001 Mar 18;69(3):89-95. doi:10.1067/mcp.2001.113989

70. Martin DC. The Mental Status Examination. In: Walker HK, Hall WD, Hurst JW, editors. Clinical methods: the history, physical, and laboratory examinations 3rd edition [Internet]. Boston: Butterworths; 1990 [cited 2025 Jul 10]. Chapter 207. Available from: https://www.ncbi.nlm.nih.gov/books/NBK320/

71. National Collaborating Centre for Infectious Diseases. More than just numbers: exploring the concept of “burden of disease” [Internet]. National Collaborating Centre for Infectious Diseases. 2016 [cited 2025 Jul 11]. Available from: https://nccid.ca/publications/exploring-the-concept-of-burden-of-disease/

72. Wimmer BC, Bell JS, Fastbom J, Wiese MD, Johnell K. Medication regimen complexity and polypharmacy as factors associated with all-cause mortality in older people: a population-based cohort study. Ann Pharmacother. 2015 Dec 17;50(2):89-95. doi:10.1177/1060028015621071

73. Gerber NL, Price JK. Chapter 21 - measures of function and health-related quality of life. In: Gallin JI, Ognibene FP, Johnson LL, editors. Principles and practice of clinical research [Internet]. Academic Press; 2017 [cited 2025 Jul 11]. Available from: https://www.sciencedirect.com/book/9780128499054/principles-and-practice-of-clinical-research#book-info

74. Centers for Disease Control and Prevention. HRQOL concepts [Internet]. archive.cdc.gov. [updated 2022 Aug 4; cited 2025 Jul 11]. Available from: https://archive.cdc.gov/#/details?url=https://www.cdc.gov/hrqol/concept.htm

75. Burkhart PV, Sabaté E. Adherence to long-term therapies: evidence for action. J Nurs Scholarsh. 2003 Sep;35(3):207. doi:10.1111/j.1547-5069.2003.tb00001.x

76. Short SE, Mollborn S. Social determinants and health behaviors: conceptual frames and empirical advances. Curr Opin Psychol. 2015 Oct;5:78-84. doi:10.1016/j.copsyc.2015.05.002

77. Civan A, Skeels MM, Stolyar A, Pratt W. Personal health information management: consumers' perspectives. AMIA Annu Symp Proc. 2006;2006:156-60

78. Centers for Disease Control and Prevention. What is health literacy? [Internet]. CDC. 2024 [cited 2025 Jul 11]. Available from: https://www.cdc.gov/health-literacy/php/about/?CDC_AAref_Val=https://www.cdc.gov/healthliteracy/learn/index.html

79. Hibbard JH, Greene J. What the evidence shows about patient activation: better health outcomes and care experiences; fewer data on costs. Health Aff (Millwood). 2013 Feb;32(2):207-14. doi:10.1377/hlthaff.2012.1061

80. American Psychological Association. Social support. In: APA Dictionary of Psychology [Internet]. [updated 2018 Apr 19; cited 2025 Jul 11]. Available from: https://dictionary.apa.org/social-support

81. Langford CP, Bowsher J, Maloney JP, Lillis PP. Social support: a conceptual analysis. J Adv Nurs. 1997 Jan;25(1):95-100. doi:10.1046/j.1365-2648.1997

82. Holt-Lunstad J, Steptoe A. Social isolation: an underappreciated determinant of physical health. Curr Opin Psychol. 2022 Feb;43:232-237. doi:10.1016/j.copsyc.2021.07.012

83. Smith KJ, Victor C. Typologies of loneliness, living alone and social isolation, and their associations with physical and mental health. Ageing and Society. 2019;39(8):1709-1730. doi:10.1017/S0144686X18000132

84. Singer C. Health effects of social isolation and loneliness. Journal of Aging Life Care. 2018;28(1):4-8. Available from: https://www.aginglifecare.org/common/Uploaded%20files/Journal%20Files/ALCA%20Journal%20Spg18_FINAL.pdf#page=4

85. Ungvarsky J. Housing stability [Internet]. EBSCO. 2024 [cited 2025 Jul 29]. Available from: https://www.ebsco.com/research-starters/politics-and-government/housing-stability

86. National Cancer Institute. Stress. In: NCI Dictionary of Cancer Terms [Internet]. [cited 2025 Jul 11]. Available from: https://www.cancer.gov/publications/dictionaries/cancer-terms/def/stress

87. Bailey RR. Goal setting and action planning for health behavior change. Am J Lifestyle Med. 2017 Sep 13;13(6):615-618. doi:10.1177/1559827617729634

88. Genewick J. Setting SMART goals for success [Internet]. Mayo Clinic Health System. 2022 [cited 2025 Jul 11]. Available from: https://www.mayoclinichealthsystem.org/hometown-health/speaking-of-health/setting-smart-goals

89. American Psychological Association. Caregiver burden. In: APA Dictionary of Psychology [Internet]. 2025 [updated 2018 Apr 19; cited 2025 Jul 15]. Available from: https://dictionary.apa.org/caregiver-burden

90. American Psychological Association. Positive aspects of caregiving [Internet]. American Psychological Association. 2011 [cited 2025 Jul 15]. Available from: https://www.apa.org/pi/about/publications/caregivers/faq/positive-aspects

91. Lawton MP, Moss M, Kleban MH, Glicksman A, Rovine M. A two-factor model of caregiving appraisal and psychological well-being. J Gerontol. 1991 Jul 1;46(4):P181-9. doi:10.1093/geronj/46.4.p181

92. Beach SR, Schulz R, Yee JL, Jackson S. Negative and positive health effects of caring for a disabled spouse: longitudinal findings from the caregiver health effects study. Psychol Aging. 2000 Jun;15(2):259-71. doi:10.1037//0882-7974.15.2.259

93. Fisher WA, Fisher JD. Understanding and promoting sexual and reproductive health behavior: theory and method. Annu Rev Sex Res. 1998;9(1):39-76. doi:10.1080/10532528.1998.10559926

94. US Department of Veterans Affairs. VA Operations Board Executive Decision Memorandum – Endorsement of the Well-Being Definition. June 20, 2024.

95. Vogt D, Borowski S, Etingen B, Merker VL, Bokhour B, Kligler B. Using well-being measurements to enhance clinical practice: why and how to ask patients about their broader well-being. Med Care. 2024;62(12 Suppl 1):S73-S75. doi:10.1097/MLR.0000000000002072

96. Pierce PF, Hicks FD. Patient decision-making behavior: an emerging paradigm for nursing science. Nurs Res. 2001 Sep-Oct;50(5):267-74. doi:10.1097/00006199-200109000-00003

97. Griffith BA, Dunham EB. Working in teams: moving from high potential to high performance. 55 City Road, London: SAGE Publications, Inc.; 2015. Chapter 3, Interpersonal dynamics and conflict; p. 43-66. doi:10.4135/9781506300153.n3

98. ProfitTrust. Cost avoidance vs cost savings: what is the difference? [Internet]. ProfitTrust. 2022 [cited 2025 Jul 15]. Available from: https://www.profit-trust.com/blog/cost-avoidance-vs-cost-savings-what-is-the-difference/

99. Bai G, Zare H. Hospital cost structure and the implications on cost management during COVID-19. J Gen Intern Med. 2020 Jun 30;35(9):2807-2809. doi:10.1007/s11606-020-05996-8

100. Centers for Disease Control and Prevention. Cost-effectiveness analysis [Internet]. CDC. 2024 [cited 2025 Jul 15]. Available from: https://www.cdc.gov/polaris/php/economics/cost-effectiveness.html?CDC_AAref_Val=https://www.cdc.gov/policy/polaris/economics/cost-effectiveness/index.html

101. U.S. Department of Veterans Affairs. Reimbursed VA travel expenses and mileage rate [Internet]. VA.gov. [updated 2025 June 30; cited 2025 Jul 15]. Available from: https://www.va.gov/resources/reimbursed-va-travel-expenses-and-mileage-rate/

102. U.S. Department of Veterans Affairs. Current VA health care copay rates [Internet]. VA.gov. [updated 2025 June 30; cited 2025 Jul 15]. Available from: https://www.va.gov/health-care/copay-rates/

103. Mitchell RJ, Bates P. Measuring health-related productivity loss. Popul Health Manag. 2011 Apr 13;14(2):93-8. doi:10.1089/pop.2010.0014
